# Organelle-targeted therapies: a comprehensive review on system design for enabling precision oncology

**DOI:** 10.1038/s41392-022-01243-0

**Published:** 2022-11-19

**Authors:** Jingjing Yang, Anthony Griffin, Zhe Qiang, Jie Ren

**Affiliations:** 1 Institute of Nano and Biopolymeric Materials, School of Materials Science and Engineering, Tongji University, 201804 Shanghai, China; 2 School of Polymer Science and Engineering, University of Southern Mississippi, Hattiesburg, MS 39406 USA

**Keywords:** Drug development, Drug development

## Abstract

Cancer is a major threat to human health. Among various treatment methods, precision therapy has received significant attention since the inception, due to its ability to efficiently inhibit tumor growth, while curtailing common shortcomings from conventional cancer treatment, leading towards enhanced survival rates. Particularly, organelle-targeted strategies enable precise accumulation of therapeutic agents in organelles, locally triggering organelle-mediated cell death signals which can greatly reduce the therapeutic threshold dosage and minimize side-effects. In this review, we comprehensively discuss history and recent advances in targeted therapies on organelles, specifically including nucleus, mitochondria, lysosomes and endoplasmic reticulum, while focusing on organelle structures, organelle-mediated cell death signal pathways, and design guidelines of organelle-targeted nanomedicines based on intervention mechanisms. Furthermore, a perspective on future research and clinical opportunities and potential challenges in precision oncology is presented. Through demonstrating recent developments in organelle-targeted therapies, we believe this article can further stimulate broader interests in multidisciplinary research and technology development for enabling advanced organelle-targeted nanomedicines and their corresponding clinic translations.

## Introduction

Cancer-related mortality remains to be an ongoing monumental global crisis, with an estimated 19.3 million new cases and 10 million cancer deaths worldwide (9.9 million excluding nonmelanoma skin cancer) in 2020, according to the International Agency for Research on Cancer.^1–3^ Developing highly efficient and precise cancer theranostics is an extremely important research area for public health and society development. Notably, nanomaterials have garnered substantial attention in cancer theranostics due to their superior performance in pharmacokinetics/pharmacodynamics (PK/PD), side-effect reduction and ease of formulation with multi-functionalities.^4–6^ However, approximately only 0.7% of administrated nanomedicines could reach their final (sub)cellular targets due to physiological and pathological barriers, and these nanomedicines often exhibit severely compromised efficacy.^7,8^ Furthermore, the clinical application of nanomedicines is often hindered by risks of low bio-availability, amplified dose, and rejection effects.^9^

**Fig. 1:**
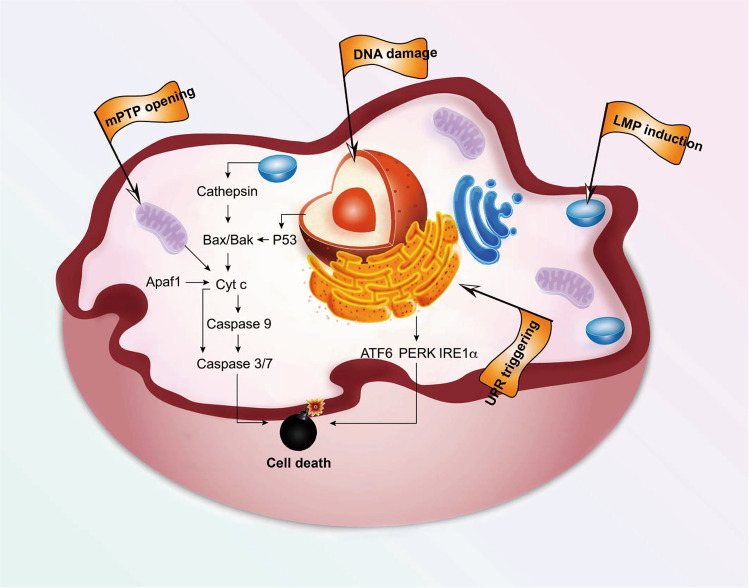
Organelle-targeted therapy boosts cancer treatment outcomes by allowing for maximum accumulation of therapeutic agents in targets, triggering specific cell death pathways

Precision medicine, or personalized medicine, offers the potential for best-practice interventions in cancer treatment with subcellular organelles representing ideal targets.^10–12^ In the era of precision medicine, targeting molecular-based pathogenesis becomes an established paradigm of cancer therapeutic agent development.^13,14^ Organelle-targeted therapies focused on the highly-sensitive and precise attack on specific organelles have received substantially growing research interest (Fig. 1).^15^ These strategies can accurately regulate the transport processes of therapeutic agents from the plasma membrane to action targets, boosting drug efficiency while maintaining necessary concentrations to induce apoptosis.^16^ Therefore, organelle-targeted strategies hold great potential to overcome physiological and pathological barriers, greatly decrease therapeutic threshold dosage, minimize side effects and, ultimately, boost treatment outcomes.

Subcellular-targeting strategies are a very promising cancer modality, while the structures and death-induced modes of organelles still remain elusive. Additionally, transport of nanomaterials to organelle targets can be restricted by cell crowding and complex cell environments, such as cytoskeletal structures.^17^ Therefore, a subtler design of organelle-targeted nanoplatforms based on unique organelle characteristics is desired to meet demands of varying cancer treatment modalities, allowing the ability to achieve maximized treatment efficacy.^18^

Subcellular organelles, such as the nucleus, mitochondria, lysosome and endoplasmic reticulum (ER), can maintain a balance between cell proliferation and death, while modulating cell metabolism functions.^19^ This article will primarily focus on discussing these essential therapeutic targets for advanced cancer treatments. We will introduce target structures, intracellular organelle functions, organelle-mediated cell death and intervention mechanisms, and design guidelines for advanced organelle-targeted cancer nanosystems. We also aim to provide a comprehensive review on the efficacy of cancer therapy methods based on subcellular organelle-specific oncology, which is vital in revolutionizing treatments and enabling the curing of cancer.

## Key milestones in developing organelle-targeted therapies

**Fig. 2:**
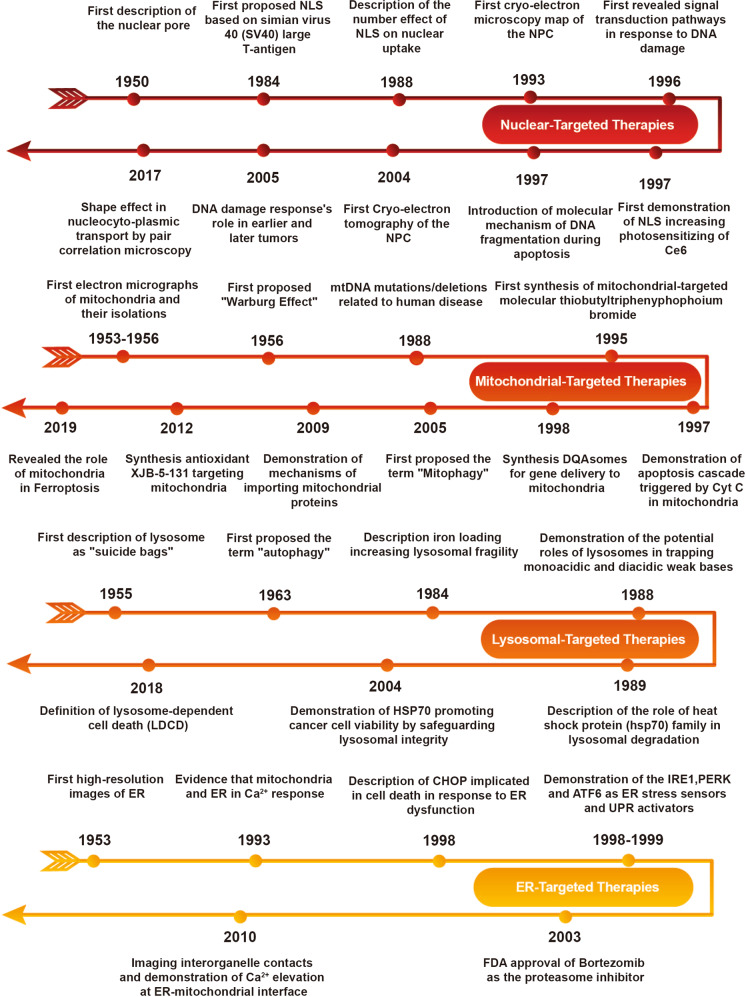
Timeline of the key advances in developing organelle-targeted therapies

Organelle-targeted therapies, which seek to enhance the efficacy of therapeutic agents, have been studied for nearly 70 years. These strategies are built on the basis of organelle spatial structure analysis, organelle-mediated death signaling pathway investigation, and therapeutic agent development. Herein, this section focuses on discussing critical studies in the development of organelle-targeted therapies in cancer treatment and outline these hallmark events in Fig. 2.

### Nucleus-targeted therapy

The nuclear pore plays an essential role in bi-directional nucleocytoplasmic transport, first described in 1950.^20^ In 1993 and 2004, the first cryo-electron microscopy map and tomography of nuclear pore complexes were obtained, respectively. These important milestones were instrumental in determining the nucleocytoplasmic transportation mechanism for macromolecules.^21,22^ With continuous, extensive research efforts focusing on elucidating nuclear structure, transport of macromolecules between the cytoplasm and nucleus was progressively recognized and then widely accepted. In 1984, Kalderon et al.^23^ first proposed nuclear localization signals based on the simian virus 40 (SV40), which assists macromolecules across the nuclear envelope (NE). Notably, the earliest research of nuclear-targeted photodynamic therapy (PDT) was in 1997 by Akhlynina.^24^ Since then, much effort has been made to understand transport mechanisms, which drives nucleus-targeted therapy development.^24–27^ Concurrently, there has been growing attention to signal transduction pathways in response to DNA damage, which facilitated more comprehensive investigations into nuclear-targeted strategies.^28–30^

### Mitochondrial-targeted therapy

Mitochondria are essential organelles that generate most of energy supply for cells, control metabolic pathways, and regulate cell death. Early critical research in determining mitochondrial structure was conducted from 1953 to 1956.^31–33^ Since then, mitochondrial-targeted molecules have facilitated antioxidant mitochondrial accumulation based on their unique structure.^34–37^ Additionally, great efforts have been delivered to understand cell death caused by mitochondrial. Research focused on mitochondria-mediated death signaling pathways have received significant attention since when the “Warburg Effect” was first proposed in 1956.^38^ Multiple lines of evidences have implicated mitochondrial dysfunction, such as apoptosis.^39,40^ Mitophagy and ferroptosis were first proposed in 2005 and 2019, respectively.^41,42^ In general, personalized therapeutic strategies toward mitochondria is an important research area in cancer treatment.

### Lysosomal-targeted therapy

Since the first discovery of lysosomes in 1955 by Christian de Duve, research investigations into lysosome structure-function relationships has led to significant progress in obtaining a deep understanding.^43^ In 1963, following the groundbreaking study of morphological processes of lysosomes under electron microscopy, Christian de Duve coined the term “autophagy.”^44^ In 1984, Glaumann et al.^45^ revealed that iron overload can result in increased lysosomal fragility,^45^ inspiring various studies on lysosome-mediated cell death. In 2018, cell death triggered by lysosomes was officially termed lysosome-dependent cell death (LDCD) by Nomenclature Committee on Cell Death.^46^ However, a study published in 1989 found that the heat shock protein (hsp70) family can regulate intracellular protein degradation in lysosomes.^47^ In 2004, Gyrd-Hansen et al.^48^ found that hsp70 promoted cancer cell viability by stabilizing the lysosomal membrane. The safeguarding function of hsp70 predicts poor therapy efficacy in lysosome-targeted therapies; thus, hsp70 is a potential target to enhance LDCD sensitivity and induce cell death.

### ER-targeted therapy

In 1953, the first high-resolution images of the ER were successfully obtained.^49^ Since then, many research studies have enabled a deep understanding about ER functions, such as the relationship between the ER and cell death; unfolded protein response (UPR) under ER stress occupies a pivotal position in cell death. In the late 1990s, IRE1, PERK, and ATF6 were reported as ER stress sensors and UPR activators to safeguard ER functions or trigger ER-mediated cell death signaling pathways.^50–53^ With improved understandings of the UPR mechanism, many proteasome inhibitors were developed to trigger cancer cell death. For example, bortezomib was approved by FDA for cancer treatment in 2003.^54^ Moreover, understandings about the relationship between the ER and mitochondria in Ca^2+^ response was first obtained in 1993^55^ and then enhanced in 2010,^56^ which provided important fundamental insights to provide new therapeutic avenues for ER-targeted therapeutic agent development.

Organelle-targeted therapeutic agents are experiencing an explosive growth in research and technology development as a very promising treatment strategy, with the ability to precisely attack specific molecular targets. It is anticipated that organelle-targeted strategies will continue to attract growing interests and very likely become a pillar of precision medicine in future cancer treatment.

## Nucleus-targeted strategies for precision attack life blueprint

### Structural components of nucleus

The nucleus has been considered as the fundamental, functional building block of the cell in regards to activity regulation, including proliferation, apoptosis, and metabolism.^57^ However, the structure of the nucleus remains elusive and has been a subject of lively debate. The nucleus, in many cases, was believed to have a nucleoskeleton, while people also proposed that it had no more than a transient complex and a membrane-bound bag of genetic materials.^58^ With exciting development in cryo-electron microscopy, X-ray crystallography, and computer-aided proteomic techniques, significant advances in understanding the nuclear structure and nucleocytoplasmic transport pathways have been achieved in recent years.^59,60^ These findings suggest that the nucleus has its own distinct substructure that acts as a dynamic organelle, rather than a rigid framework.^60^

The nucleus, in eukaryotic cells, is enclosed by a double membrane separating the nucleoplasm and cytoplasm, termed as nuclear envelope (NE).^58^ As a compartment border, the NE ensures versatile communication, secured macromolecule exchange between nucleus and cytoplasm, and genetic material safeguarding. Moreover, the NE is highly adaptable and dynamic, which can be reflected by its disassembly and reformation during mitosis, composition fluctuations during differentiation and deformation, and transient rupture or repairment under mechanical pressure.^61^ The double membrane is designated as the inner nuclear membrane (INM) and the outer nuclear membrane (ONM), which are essential components in transporting millions of molecules per second in bi-directional nucleocytoplasmic trafficking.^62^

Nucleocytoplasmic exchange processes are regulated by nuclear pore complexes (NPCs), which are approximately 30–50 nm in diameter and 50–80 nm in length, whose central channels perforate the NE as a bridge.^63,64^ The NPC is a highly modular symmetrical scaffold, composed of an eightfold symmetrical ring and spoke assembly, cytoplasmic fibers, and a filamentous nuclear basket.^65,66^ Furthermore, more than 500 copies of 30 different nucleoporins (nups) proteins are conserved with biochemical stability, arranged within the building blocks of NPCs.^67^ A specific phenylalanine-glycine nucleoporin (FG Nups) harbors intrinsically disordered FG-rich domains, which occupied the central channel of NPCs to achieve selective transport within milliseconds.^68,69^ Nucleocytoplasmic transport exhibited a robust profile under the assist of FG Nups, however, the physical properties of cargoes exert a non-negligible impact on the efficiency in nucleocytoplasmic trafficking.^70^ Relatively small cargoes can permeate freely through NPCs, while large macromolecules are impeded. These large cargoes can only achieve rapid transport through NPCs with the aid of karyopherin and FG Nups interaction.

Bi-directional nucleocytoplasmic trafficking with high efficiency and selectivity in complex and crowded conditions is enabled by the highly-organized sub-units of the nucleus, coordinating with one another.^71^ Elaborate structures of the NPC allow macromolecules to perform precise and efficient shuttling between the nucleus and cytoplasm for contributing to cellular homeostasis. The malfunction of nucleocytoplasmic trafficking can lead to protein mis-localization and directly affect gene expression, signal transduction, and diseases.^72^ Therefore, understanding the organizing framework of the nucleus, including the substructure inside cell nuclei as well as how the nucleocytoplasmic transport is coordinated and regulated, is the essential basis for nuclear-targeted transportation.

### From DNA damage to cell death

The nucleus is the most prominent organelle of the eukaryotic cell, which serves as the container of the majority of cellular genetic information and coordinates gene expression.^73,74^ Genome integrity is paramount to life as irrevocable damage to nuclear DNA (nDNA) can adversely impact cellular function, viability, and growth. However, thousands of DNA lesions are constantly attacked per day.^75^ To stay alive, the ability of an individual cell to act appropriately is important, especially when their genome is threatened by an intrinsic or extrinsic insult.^76^ If damage is too severe or the attempted repair is ineffective, the DNA damage response (DDR) can trigger a proapoptotic signaling cascade and initiates an apoptotic response.^77,78^ Notably, DDR defects are a pervasive hallmark of cancer cells, causing detrimental mutation accumulation.

Double-strand breaks (DSBs), which is the most severe type of DNA damage, occur when the phosphodiester backbone is disrupted on both strands.^76^ Most DNA-damaging agents can cause DSBs to trigger apoptosis.^79^ DSBs are considered to be a crucial initiator of the apoptosis signaling pathway.^80^ Ataxia telangiectasia mutated kinase (ATM), Rad3-related, and ataxia telangiectasia-related (ATR) kinases, which initiate DNA damage checkpoints and DDR, are activated upon DSBs. Subsequently, additional checkpoint kinases, Chk1 and Chk2, are phosphorylated by ATM and ATR kinases and act downstream to activate p53. P53 then transactivates pro-apoptotic genes through transcription, such as Bax, Fas, and Puma, and induces apoptosis. Furthermore, DNA damage-mediated cell death is not solely regulated through genome regulation as complex enzymatic reactions also play an important role.^74^ Cells intricately respond to DSBs by evoking related signaling pathways that may ultimately trigger DNA repair or initiate cell death-related pathways to eliminate the damage.^80^ Uncovering the relationship between DNA damage and cell death can allow a deep understanding about the pathogenesis of cancer as well as the development of more effective therapies.

### DNA intervention strategies

Irreversible damage to DNA is a key driving force of cell death perturbation. Common DNA intervention mechanisms either arrest transcription, lesion DNA repairing processes, or block DNA replication.^81^ Therefore, it is apparent that DNA intervention is a crucial mechanism in apoptosis, exerted by many toxins in cancer treatment (Table 1). A strategy of great interest and potential is to enhance cancer cell apoptosis efficiency of toxins by amplifying DNA damage.

**Table 1:** Overview of organelle-targeted therapeutic agents applied in cancer treatments

Organelle	Targeted factors	Toxins	Intervention mechanism	Therapy	Others	Models	Ref.
Nucleus	NLS peptide	CPT prodrug	DNA damage	Chemotherapy		In vitro	^407^
	TAT/RGD peptide	DOX/CS-6@ Erythrocyte membrane-encapsulated GOQDs	DNA damage/VEGF expression ↓	Chemotherapy		In vivo	^87^
	TAT peptide	DOX@MSNs	DNA damage	Chemotherapy	Overcoming MDR	In vitro	^408^
	TAT peptide	CPT@FMSN	DNA damage	Chemotherapy	Charge-reversal strategy	In vitro	^409^
	Passive targeting	DOX@PELA micelles	DNA damage	Chemotherapy	Variable-size changeable strategy	In vivo	^410^
	R8NLS peptide	H1 peptide@HPMA polymer	Interference c-Myc DNA binding	Chemotherapy	Multi-stage targeting strategy	In vivo	^411^
	NLS peptide	Gold nanorods	DNA damage	PTT		In vivo	^94^
	Passive targeting	Hf-HI-4COOH polymer	DNA damage	PTT	Low-temperature thermal ablation	In vivo	^412^
	Passive targeting	Ir-Es complexes	Nuclear damage	PDT	Irradiation improvement	In vivo	^413^
	TAT peptide	Ce6@Upconversion-TiO_2_ Nanoparticles	DNA replication disruption	PDT	Irradiation improvement	In vivo	^414^
	NLS peptide	PpIX@Chimeric peptide nanoparticles	DNA damage	PDT	Charge-reversal strategy	In vivo	^415^
	AS1411 aptamer	Ce6@MOF nanoenzyme	DNA damage	PDT	Overcoming hypoxia	In vivo	^416^
	NLS/CPP/RGD peptide	ASO@PyTPE	DNA interference/Bcl-2 ↓	Gene therapy		In vitro	^417^
	RGD-R8-PEG-HA	pDNA@Fluorinated polymer PF33	DNA interference	Gene therapy	Multi-stage targeting strategy	In vivo	^418^
Mitochondria	TPP	Resveratrol Prodrug	Metabolism disruption	Chemotherapy		In vitro	^419^
	TPP	ATO/DOX Prodrug	mtDNA damage/MMP↓	Chemotherapy	Redox-responsive strategy	In vivo	^420^
	TPP	DOX@Self-assembled cyanostilbene nanoparticles	Unbalance of redox states/MMP↓	Chemotherapy		In vivo	^421^
	Organometallic rhenium complexes	DOX	Metabolism disruption/topoisomerase II ↓	Chemotherapy	Overcoming MDR	In vitro	^215^
	TPP	Cisplatin@PLGA-*b*-PEG nanoplatforms	mtDNA damage	Chemotherapy	Overcoming MDR	In vivo	^422^
	PPh3	SOPs	Mitochondrial dysfunction	PTT	ATOT strategy	In vivo	^423^
		Single-walled carbon nanotubes-chitosan nanoparticles	MPTP	PTT	Charge-reversal strategy	In vitro	^424^
		ATO/IR820 @ Self-assembled nanodrugs	Metabolism disruption/HSPs expression ↓	PTT	Overcoming thermotolerance	In vivo	^425^
	CTPP	CAT/Ce6 @ hollow silica nanoparticles	Metabolism disruption/unbalance of redox states/mtDNA damage	PDT	Overcoming hypoxia	In vivo	^426^
	CyNH2	Acetylated lysine- CyNH2	MMP↓/unbalance of redox states	PDT	Imaging-guided PDT	In vivo	^427^
		UCNP@TiO_2_ NCs	Unbalance of redox states/metabolism disruption	PDT	Irradiation improvement	In vivo	^428^
	Ir(III) complexes	Ir(III) complexes	Unbalance of redox states/metabolism disruption	PDT	Irradiation improvement	In vitro	^429^
	MLS	pDNA@DQAplexes	Initiation mtDNA transcription	Gene therapy		In vitro	^210^
	R8 moieties	ASO@MITO-Porter system	Metabolism disruption	Gene therapy		In vitro	^430^
Lysosomes	Endocytosis	Iron(III)-activated iridium(III) prodrug	Inducing Fe releasing/LMP	Chemotherapy		In vivo	^431^
	Endocytosis	PTX@PDA coated MS NPs	LMP	Chemotherapy		In vivo	^432^
	Endocytosis	DOX@Sericin protein modified MSNs	LMP	Chemotherapy	Overcoming MDR	In vivo	^433^
	Morpholine and cRGD	Morpholine-cRGD peptide	LMP	PTT		In vivo	^434^
	FA	Ruthenium nitrosyl donor	LMP	PDT	NO and ROS synergistic PDT	In vitro	^435^
	Endocytosis	BDP-688/P16FP@ Polymer micelles	LMP	PDT	Imaging-guided PDT	In vivo	^436^
	Endocytosis	BODIPY dyes@Polymer micelles	LMP	PDT	PAI-guided PDT	In vivo	^437^
Nucleus-Mitochondria-Lysosomes	Endocytosis	platinum-doped carbon nanoparticles	Thermal damage to multi-organelles	PTT	Multiple-organelle synergistic strategy	In vivo	^438^
Mitochondria-Lysosomes	Endocytosis and TPP	AIE-Mito-TPP & AlPcSNa4	LMP/metabolism disruption/ unbalance of redox states /mtDNA damage	PDT	Multiple-organelle synergistic strategy	In vivo	^439^

*ASO* antisense oligonucleotide, *ATO* arsenic trioxide, *ATOT strategy* active tumor- and organelle-targeted theranostic strategy, *AIE* aggregation-induced emission, *CPT* camptothecin, *CS-6* gamabufotalin, *Ce6* chlorin e6, *CAT* catalase, *CPP* cell-penetrating peptide, *DOX* doxorubicin, *DQAplexes* cationic ‘bola-lipid’-based vesicles, *FMSN* folic acid modified magnetic mesoporous silica nanoparticles, *GOQDs* graphene oxide quantum dot,*HPMA polymer* N-(2-hydroxypropyl) methacrylamide polymer, *Hf-HI-4COOH polymer* heptamethine cyanine dye-based nanoscale coordination polymer, *Ir-Es complexes* terpyridine-based cyclometalated Iridium(iii) complexes, *LMP* lysosomal membrane permeabilization, *MSNs* mesoporous silica nanoparticles, *MDR* multidrug resistance, *MMP* mitochondrial membrane potential, *MOF* metal-organic framework, *NLS* nuclear localization signal, *PELA micelles* mPEG-PLA-ss-PEI-DMMA polymer micelles, *PpIX* alkylated protoporphyrin IX, *pDNA* plasmid DNA, *PAI* photoacoustic/optoacoustic imaging, *PTT* photothermal therapy, *PDT* photodynamic therapy, *ROS* reactive oxygen species, *SOPs* small-molecule organic photothermal agents, *TPE* two-photon excitation, *UCNP* upconversion nanoparticle, *VEGF* vascular endothelial growth factor

#### Chemotherapeutic agents

DNA-damaging chemotherapeutic agents, such as cisplatin and doxorubicin (DOX), are widely used in chemotherapy, where they can interfere with DNA replication and transcription. Cisplatin cytotoxicity, for example, is caused by the formation of interstrand, or intrastrand, adducts with DNA, which destroys DNA function and induces irreparable DNA lesions.^82,83^ Another typical DNA toxin, DOX can intercalate into stranded DNA to form DNA adducts for increasing torsional stress. Moreover, DOX inhibits enzyme topoisomerase II, thus preventing DNA replication and inducing DNA breaks.^84^ In addition, several molecule inhibitors, such as Elimusertib, Prexasertib, and PHI-101, have been developed to disrupt DDR signaling pathways, thus promoting cancer cell death. As shown in Table 2, many nuclear-targeted therapeutic agents are currently under clinical trials.

**Table 2:** Summary of organelle-targeted cancer therapies under clinical trials

Drug name	Intervention mechanism	Cancer type	Clinical Trials.gov Identifier	Phase	Ref.
Elimusertib (BAY1895344)	ATR inhibition	Advanced solid tumors, lymphomas	NCT03188965	Phase 1	
Elimusertib, Niraparib	ATR inhibition, PARP inhibition	Advanced solid tumors (excluding prostate cancer), ovarian cancer	NCT04267939	Phase 1	
AZD6738	ATR inhibition	Solid tumor refractory to conventional treatment	NCT02223923	Phase 1	
Veliparib, VX-970, Cisplatin	PARP inhibition, ATR inhibition	Neoplasms	NCT02723864	Phase 1	
Azd6738, Olaparib	ATR inhibition, PARP inhibition	Gynecological cancers	Nct04065269	Phase 2	
Atg-018	ATR inhibition	Advanced solid tumors, hematological malignancies	Nct05338346	Phase 1	
Prexasertib (Ly2606368),	CHK1 inhibition	Advanced solid tumors	NCT02873975	Phase 2	
Prexasertib (Ly2606368), Olaparib	CHK1 inhibition, PARP inhibition	Solid tumor	NCT03057145	Phase 1	
LY2880070, Gemcitabine	CHK1 inhibition	Ewing sarcoma, Ewing-like sarcoma	NCT05275426	Phase 2	
SRA737	CHK1 inhibition	Advanced solid tumors or non-Hodgkin’s lymphoma	NCT02797964	Phase 1 Phase 2	
PHI-101	CHK2 inhibition	Platinum-resistant ovarian cancer, platinum-refractory ovarian carcinoma, platinum-resistant fallopian tube carcinoma, platinum-resistant primary peritoneal carcinoma	NCT04678102	Phase 1	
Talazoparib (BMN 673)	PARP inhibition	Advanced ovarian cancer, primary peritoneal cancer, advanced breast cancer, advanced solid tumors	NCT01989546	Phase 1 Phase 2	
Ceralasertib	DDR intervention	Head and neck squamous cell carcinoma	NCT03022409	Phase 1	
Lmp400	DNA damage, delays DNA repair	Neoplasm, lymphoma	NCT01794104	Phase 1	
Metformin	Metabolism disruption	Brain neoplasms	NCT02149459	Phase 1	
Temozolomide	Metabolism disruption	Lung cancer	NCT00022711	Phase 2	^440^
Dichloroacetate (DCA)	Mitochondrial pyruvate dehydrogenase kinase inhibition	Malignant gliomas, glioblastoma multiforme	NCT00540176	Phase 2	
Ketoconazole (Kcz) Posaconazole (Pcz)	HK2 inhibition	High-grade gliomas (WHO grade III and IV)	NCT03763396	Early Phase 1	
ARQ 501	Redox homeostasis disruption	Head and neck neoplasms, carcinoma, squamous cell	NCT00358930	Phase 2	^441^
Motexafin gadolinium	Redox homeostasis disruption	Renal cell carcinoma	NCT00134186	Phase 2	^442^
Pantoprazole	Proton pump inhibition	Advanced solid tumors	NCT01163903	Phase 1	
Omeprazole	Proton pump inhibition	Breast cancer	NCT02595372	Phase 2	
Lansoprazole	Proton pump inhibition	Breast cancer	NCT04874935	Phase 3	
Bortezomib	Blocking the enzymes necessary for cancer cell growth	Recurrent breast cancer, stage IV breast cancer	NCT00025584	Phase 2	
Nelfinavir	AKT signaling inhibition	Colorectal cancer	NCT00704600	Phase 2	
Belinostat Atazanavir	Proteasome inhibitors	Hematological malignancies	NCT04184869	Phase 1	

*ATR* Ataxia-telangiectasia and Rad3 related protein, *CHK1* checkpoint kinase 1, *DDR* DNA damage response, *PARP* poly (ADP-ribose) polymerase, *HK2* hexokinase 2

The efficiency of chemotherapeutic drugs acting on DNA depends on the pharmacological effective drug concentration at the nuclear site. However, it is pertinent to point out that only ~1% of cisplatin and ~0.4% of Dox could pass through intracellular barriers and reach the nucleus in a pharmacological concentration.^85,86^ Notably, successful transport to the nucleus utilizing a nuclear-targeted strategy would significantly boost therapeutic outcomes as the damage in the nucleus is destructive to cells. As an example, Fan et al.^87^ constructed biomimetic nanocarriers (GTDC@M-R) based on erythrocyte membrane-encapsulated graphene oxide quantum dots (GOQDs) for DOX and CS-6 delivery. TAT and RGD peptides were attached to the surface of GOQDs and erythrocyte membranes to achieve dual-targeting of the nucleus and triple-negative breast cancer (TNBC) cell membranes. In this study, Gamabufotalin (CS-6) markedly reduced aggressiveness in TNBC via down-regulation of vascular endothelial growth factor (VEGF) expression and inhibited angiogenesis. As a result, GTDC@M-R regulated the signaling pathway of apoptotic (BAX/Bcl-2 and p53) and metastasis (COX-2 and VEGF), which effectively suppressed tumor growth, invasion, and metastasis.

#### Thermal ablation

High temperature can inhibit DNA replication,^88–90^ due to thermal-mediated enzyme denaturation related to DNA replication (such as DNA polymerase α and β responsible for DNA replication and repair). Additionally, the aberrant condensation of nuclear matrix proteins induced by high-temperature leads to blockage of DNA replication.^90^ Collectively, the local high temperature locally at the nucleus, leads to defects in normal DNA functions and is responsible for thermal-mediated death of cells.

Nuclear-targeted thermal ablation nanoplatforms only require low-power density to achieve high-efficiency therapeutic treatment, which may be a practical approach for optimal cancer treatment.^91,92^ To date, gold nanomaterials such as nanorods, nano-stars, nanocages, and nano-shells have been employed as nuclear-targeted thermal ablation nanoplatforms due to their remarkable surface plasmon resonance (SPR) effect, high-efficiency in light-to-heat conversion, and excellent photothermal stability, which can collectively enhance the therapeutic efficacy.^93^ Pan et al.^94^ synthesized a nuclear-targeted therapeutic system (GNRs-NLS) which caused DNA damage and DNA repair process failed at low NIR intensity (0.2 W/cm^2^), resulting in apoptosis without excessive inflammation.

#### Phototoxicity

It is pertinent to note that excessive reactive oxygen species (ROS) can serially damage DNA upon lipophilic photosensitizer accumulation in the nucleus under laser irradiation, leading to single-strand breaks and inactivated DNA repair enzyme.^95,96^ Moreover, photodynamic therapy (PDT) induces destabilization of [Ca^2+^] and ROS-induced nuclear-pore expansion which directly damage the nucleus and lead to apoptosis.^97,98^ Nuclear-targeted photosensitizer chlorin (Ce6) was first developed by Akhlynina in 1997, showing that nuclear-targeted PDT enhanced therapeutic effects with EC_50_ can decrease by almost 2000-fold.^24^ Accurate bombardment has increased viability in PDT, with the nucleus as the damage-sensitive site, which demonstrates that nuclear-targeting as an effective strategy for enhancing PDT in cancer treatment.

#### nDNA expression interference

Gene therapy is a promising strategy for enabling a permanent cure in cancer research. Extraneous genes (double-strand DNA (dsDNA), single-strand DNA (ssDNA), plasmid DNA, antisense oligonucleotide, and small interfering RNA) are developed to interrupt, eliminate, or correct genetic defects and anomalies to alter endogenous gene expression.^99–101^ As of November 2017, gene therapy clinical studies were reviewed to encompass 2,597 trials within 38 countries.^102^ However, a lack of full understanding about its safety and efficiency within this rapid-developing technological area currently hinders their practical implementation. Therefore, developing innovative, safe and robust approaches to achieve accurate therapeutic agent nuclear accumulation to circumvent existing challenges is of great significance in cancer treatment.

### The future of nuclear-targeted therapies design

Nuclear translocation continues to be a complicated spatiotemporal challenge. Entry into the nucleus is considered to be regulated by the transport kinetics of NPCs.^103,104^ Deciphering the nuclear import machinery will further facilitate the construction of nuclear-targeted nanosystems.

#### Passive diffusion

**Fig. 3:**
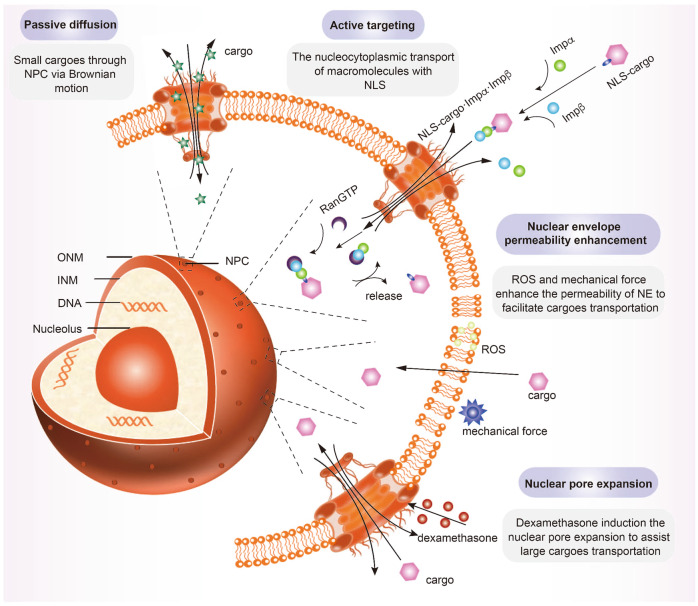
Design guidelines of nuclear-targeted nanosystems. Small cargoes cross the nuclear envelope through passive diffusion, without interaction with NPC. Active targeting is necessary for large cargoes. NLS-cargoes interact with nuclear transport receptors to achieve large cargoes accumulation in the nucleus. Nuclear envelope permeability enhancement allows ROS and mechanical forces to enhance the permeability of the nuclear envelope, and thus facilitate large cargo nuclear transportation. Nuclear pore expansion is a direct manner that dexamethasone regulates to expand the size of NPC. INM inner nuclear membrane, ONM outer nuclear membrane, NPC nuclear pore complexes, NLS nuclear localization signal, Impα importin α, Impβ importin β, RanGTP Ran guanosine triphosphate

Passive diffusion is the equilibration of relatively small cargoes, macromolecules with up to 40 kDa in molecular weight, between the cytoplasm and the nucleoplasm, which is a result of Brownian motion and without specific interaction with the NPC domain (Fig. 3).^105^ The specific size and shape of cargoes are found to be the permeant determinants of passive diffusion rates.^69^

The passive diffusion behavior of cargoes through the NPC highly depends on the size threshold. Paine et al.^106^ proposed that the patent radius of NE pores is approximately 45 Å, which can restrict molecular movement in nucleocytoplasmic transport. This observation revealed the sieving properties of NE. Since then, substantial research has been performed on size limitation in passive diffusion and it was found that passive diffusion is relatively fast for small cargoes with molecular weight in the range tof 20–40 kDa.^107^ Passive transport rates became more restricted and inefficient beyond such thresholds.

Recent in vitro studies evaluated the effect of size-dependency on permeability and intranuclear accumulation of tiopronin-covered Au nanoparticles (Au-TIOP NPs) with diameters of 2, 6, 10, and 16 nm in MCF-7 cancer cells.^108^ After 24 h, the larger Au-TIOP NPs were primarily localized in the cytoplasm. However, Au-TIOP NPs with diameters less than 10 nm could efficiently enter the nuclear. Additionally, folic acid (FA) modified carbon quantum dots (CDs) with diameters smaller than 9 nm exhibited excellent nuclear translocation efficiency in oral cancer cells.^109^ In general, cargo sizes smaller than 9 nm enable efficient and unrestricted permeation to the NE, whereas larger cargoes exhibit limited transport.

Cargo morphologies can modulate the rate and efficiency of barriers crossing during nuclear import.^110^ Gaus et al.,^27^ were inspired by the shape of pathogens, which developed nanoparticles with varying shapes (including vesicles, micelles, rods, and worms) and identical surface chemistry. Their results demonstrated that rod- and worm-shaped nanoparticles with a high-aspect-ratio tended to have higher nuclear accumulation. Therefore, high-aspect-ratio nanoparticles seem to be more promising for nuclear-specific accumulation, enabling a significant increase in concentration within the nucleus compared to spherical nanoparticles. The well-defined size and geometry of nanomaterials are key design parameters that need to be considered for designing nuclear-targeted therapeutic nanoplatforms.

#### Active targeting

Passive diffusion depends on several critical properties of nanomaterials as it is driven by a concentration gradient.^69,105^ Highly dynamic and disordered proteins inside of each NPC, such as FG Nups, can impede the nuclear entry of macromolecules larger than 9 nm in diameter (or molecular weight higher than 40 kDa).^111^ Moreover, nuclear accumulation through passive diffusion ceases as the concentration between nucleoplasm and cytoplasm reaches equilibrium.^112^ The efficiency of passive diffusion is frequently limited by several factors, as such it is not the preferred choice for nucleus targeting. Fortunately, the nuclear localization signal (NLS) facilitates transport and accumulation of macromolecules in the nucleus.^113^ It has been reported that NLS-containing cargos are actively transported into the nucleus through NPCs within 30 min.^114^

The NLS was first reported based on the simian virus 40 (SV40) large T-antigen by Kalderon et al. in 1984, and has attracted significant attention since then.^23^ The NLS sequence has been classified into classical NLS (cNLS) and non-classical NLS (ncNLS).^115,116^ The most well-characterized NLS is the cNLS, which contains a single stretch of a basic amino acid sequence of 4–8 amino acids, primarily including positively charged lysine (K) and arginine (R) residues, with the essential functional sequence of cNLS being K-(K/R)-X-(K/R).^117^ For example, the sequence of simian virus 40 large T antigen (SV40T) is PKKKRKV. For comparison, ncNLSs do not require these characteristics, such as the proline-tyrosine nuclear localization signal (PY-NLS).^118^

The import pathway of NLS cargoes is shown in Fig. 3. This nucleocytoplasmic transport is orchestrated by nuclear transport receptors, referred to as karyopherins, where “importins” and “exportins” regulate the import and export of signal-specific cargoes. Within the cytoplasm, importin α (Impα) recognizes and binds to NLS cargoes and, subsequently, forms a heterodimer complex with importin β (Impβ), which can be abbreviated as NLS-cargo∙Impα ∙ Impβ. Subsequently, Impβ explicitly interacts with the FG Nups to form the NLS-cargo∙Impα ∙ Impβ, which is localized at the nucleus. Ran guanosine triphosphate (RanGTP) then dissociates the complex by inducing spatial conformation changes of Impβ, resulting in NLS-cargo and Impα releasing into the nucleus. Finally, through the assist of RanGTP and Cse1, Impα is transported to the cytoplasm where it lies in wait for the next round of cargo transform.^119^ This process is also applicable to the non-classical nucleocytoplasmic transport pathway, where Impβ directly binds and transports ncNLS-cargos without involving impα.^120^

NLS is necessary for translocating large nanoparticles into the nucleus. In 1988 it was found that NLS-coated gold nanoparticles with a diameter of 26 nm were successfully transported across the NPC and achieved nuclear localization.^25^ These results led to the understandings that the threshold size of NLS-cargoes through the NPC was 26 nm. Up until 2001, the feasible diameter of NLS-cargoes complexes was speculated to increase by about 8 nm, due to classical nucleocytoplasmic transport principle development.^107^ In 2002, Kann et al.^121^ re-defined the threshold of cargo-receptor-gold complexes; NLS cargoes as large as 39 nm in diameter were able to across the NPC without disassembly process occurred. Moreover, it is currently under investigation that if chitosan nanoparticles with diameters varying between 25–150 nm,^122^ or polymeric nanoparticles with diameters ~234 nm,^123^ can achieve nuclear accumulation under the regulation of NLS.

It is still confounding that the NPC allows nanocarriers to traverse, whose immense sizes far exceed the maximum pore diameter of the NPC. The intranuclear accumulation of large nanoplatforms is separated from the participation of Impα/β, NLS, and RanGTP, due to the selective barrier of NPC.^124^ Interestingly, the interaction between NPCs and impα·impβ·NLS-cargos can result in NPC barrier reduction, nuclear pores, and NLS-cargo deformation.^124^ These results can be used to explain how large nanocarriers bypass the NPC barrier and enter the nucleus.

The significance of NLS in the nucleocytoplasmic transport of macromolecules is clear. Strategies involving NLS incorporation also significantly impact nuclear transport efficiency of ultra-small nanoparticles.^125,126^ Nevertheless, the electrostatic interactions between NLS and nanoplatforms may disrupt the stability of NLS, resulting in the failure of active nuclear-targeted transport.^127^ Therefore, randomized utilization NLS may not enhance the efficiency of nuclear translocation. Understanding the elaborate nucleocytoplasmic transport trafficking pathway in specific cells is vital for enabling the nuclear entry.

#### Nuclear envelope permeability enhancement

The NE, a complex double-membrane system, separates the nucleus and the cytoplasm while safeguarding nuclear compartmentalization.^128,129^ It was generally accepted that NE transient rupture only occurs during mitosis.^130,131^ However, recent studies revealed that reactive oxygen species (ROS) and mechanical forces allow the NE to dismantle in a spatiotemporally controlled manner (Fig. 3). Enhancing NE permeability by controlling nuclear compartmentalization may facilitate the nuclear entry of large nanomaterials whose dimensions exceed the NPC restrictions.

ROS are highly destructive chemicals that predominantly lead to lipid peroxidation in the membrane.^132,133^ Excessive accumulation of ROS damages phospholipids directly by affecting the fluidity and permeability of lipid bilayers, and ultimately compromises membrane integrity. More importantly, ROS may attack bio-membranes and subsequently induce various types of cell death, such as apoptosis, autophagy and ferroptosus.^134,135^ Wu et al.^136^ utilized light irradiation to stimulate ROS generation and facilitate nanoplatform nuclear entry. The nanoplatforms were fabricated with polyamine-containing polyhedral oligomeric silsesquioxane (POSS), polyethylene glycol (PEG), and rose bengal (RB), denoted as PPR NPs. Under mild-light irradiation, PPR NPs generate single oxygen species (^1^O_2_), which escapes from the endolysosomal compartment, and further accumulates near the nucleus to increase the permeability of NE. PPR NPs successfully deliver payloads that scarcely cross the NPC into the nuclei, and therefore functional payloads cause irreversible damage to cancer cells.

Nuclear envelope rupture, or the NER effect, is another mechanism that increases NE permeability and promotes large macromolecule passive migration. However, transient NER with incomplete sealing of the NE may yield exposure of DNA to the cytoplasm, which then leads to DNA damage. The NER effect can be controlled by vapor nanobubble-mediated photoporation of Au NPs.^137^ Upon laser activation, the temperature of perinuclear Au NPs rapidly increase and short-lived vapor nanobubbles (VBN) accumulate around the perinucleus. Following VBN collapse, high-pressure shock waves occur which lead to mechanical force impairments of NE, and the large nanoplatforms accumulate inside the nucleus due to the incomplete NE. Therefore, nuclear photoporation in a spatiotemporally controlled manner provides a powerful tool to achieve specific nuclear targeting therapeutics in oncology. Unlike the NLS-mediated nuclear translocation strategy, enhanced permeability of NE allows larger sized nanoparticles to enter the nucleus and be highly efficacious.

#### Nuclear pore expansion

The nucleocytoplasmic transport efficiency of large nanomaterials could be determined by inducing nuclear pore expansion, for example, under the effect of dexamethasone (Dex), which is a commonly applied synthetic glucocorticoid (Fig. 3).^138,139^ A plethora of studies have been performed to elucidate Dex-mediated behaviors of nucleocytoplasmic transport. Shahin et al.^26^ observed the possible effects of Dex on Xenopus laevis oocytes during nucleocytoplasmic transport, which were visualized by atomic force microscope (AFM). It was found that the apparent diameter of NPCs was remarkably enlarged up to almost 60 nm within 90 s after injecting Dex. Specifically, Dex induced dilation and conformational changes in NPCs within the ONM due to the triggering of an intracellular signal cascade in the nucleus.

Dilation behavior of NPCs, mediated by Dex, is of vital significance for nuclear translocation of nanoplatforms. Similar results were found in the study by Kastrup et al.^140^ where NPCs of Xenopus laevis oocytes dilated to 110 nm within minutes of Dex treatment, followed by increased expansion in the NPC with diameters up to ~140 nm after 5–11 min. Furthermore, pores up to 300 nm in diameter were also observed. Dex is highly specific and selective to glucocorticoid receptors (GR), expressed in almost every nucleus.^26^ Consequently, Dex has been employed to achieve cancer-cell-specific nuclear-targeted therapeutic agent delivery. In one of the studies performed by Ye et al.,^141^ Dex was used to modify WS2 nanocomposites to achieve precision with ROS- and thermal-sensitive subcellular organelles, causing irreversible damage to cancer cells. It is now recognized that Dex could be employed to enhance the nuclear pore expansion, assisting nuclear-targeted strategies by regulating nuclear entry behaviors and promoting nuclear translocation of macromolecules.

## Mito-bomb: mitochondrial-targeted strategies

### A brief overview of mitochondria

In the 1950s, mitochondria were first postulated to be linked to cellular bioenergetics after the Krebs cycle discovery.^142^ Following in-depth investigations into the cell biology of mitochondria, it was found that they can serve as the fundamental centers of cell death, controlling a plethora of signaling cascades.^143^ Mitochondria often participate in and orchestrate complex cellular processes, from controlling cell division and differentiation, to regulating cell growth and death.^144,145^ Their versatile functionalities are associated with mitochondrial architecture and biochemical activity.

**Fig. 4:**
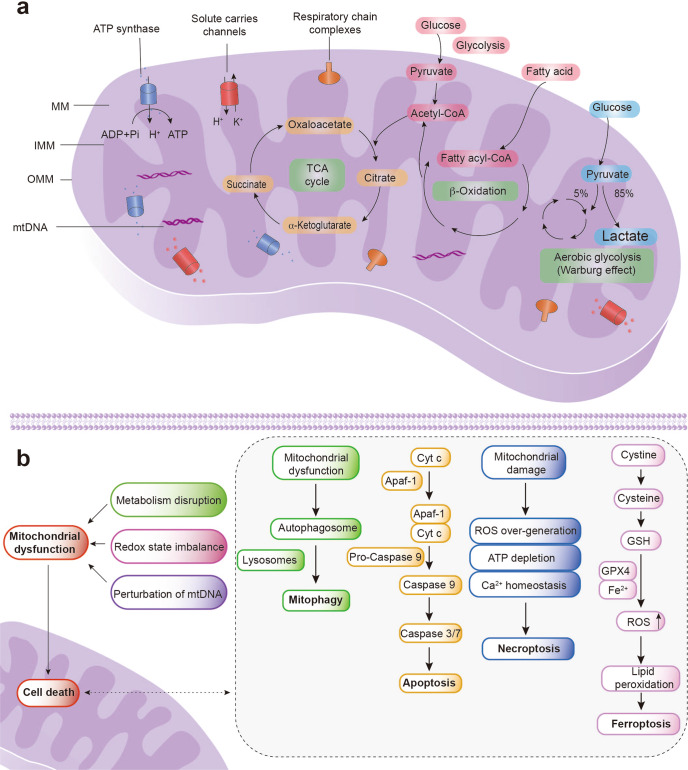
Personalized therapeutic strategies toward mitochondria. **a** The structure and function of mitochondria are displayed, with emphasis on the TCA cycle and β-oxidation. In contrast, cancer cells rely on the “Warburg effect” to achieve energy supply. **b** Mitochondrial dysfunctions to trigger cell death include metabolism disruption, redox state imbalance, and perturbation of mtDNA. Once mitochondrial damage and mPTP opening occur, cell death may occur by mitophagy, apoptosis, necroptosis, or ferroptosis. IMM inner mitochondrial membrane, OMM outer mitochondrial membrane, MM mitochondrial matrix, PDK dehydrogenase kinases, GAPDH glyceraldehyde 3-phosphate dehydrogenase, ROS reactive oxygen species, GSH glutathione, Cyt C cytochrome C

Mitochondria are defined as dynamic organelles with complex intramitochondrial compartments, including the outer mitochondrial membrane (OMM), intermembrane space (IMS), inner mitochondrial membrane (IMM), and the mitochondrial matrix (MM), as shown in Fig. 4a.^60,146^ Each intramitochondrial compartment provides unique biochemical reaction environments to maintain homeostasis and regulate metabolism.

The OMM, as the interface between the mitochondria and cytoplasm, coordinates the process of small molecule permeation and mediates the transduction of mitochondrial signals.^147^ In addition, the OMM serves as the membrane contact site to exchange constituents between the mitochondria and other organelles, including the lysosome and ER. Specific host proteins in OMM, such as translocases, can mediate mitochondrial precursor protein transport. The IMM, another mitochondrial membrane, exhibits a heavily folded structure which can be further divided into the inner boundary membrane (IBM) and mitochondrial cristae.^148^ IBM that runs in parallel to the OMM harbors high amounts of channel transporters which shuttle ions and mitochondrial respiration complexes.^149^ The IMM invaginates and forms the cristae that provides optimal surface areas for mitochondrial respiration. The cristae is a critical site for the oxidative phosphorylation pathway (OXPHOS), as it hosts various respiratory chain complexes as well as F_1_F_o_-ATP synthase.^150,151^ Moreover, cytochrome c, the caspase activator during apoptosis, can be localized at the intracristal compartment. Thus, the IMM not only participates in mitochondrial respiration and mitochondrial energy conversion, but also impacts the apoptosis.

The OMM and IMM are separated by the IMS, which acts as a critical buffer between the cytoplasm and the MM. The IMS is essential for mitochondrial metabolism and free radical scavenging, especially for maintaining cellular homeostasis.^152^ The MM is involved in metabolic reactions by regulating tricarboxylic acid, fatty acid oxidation, and amino acid metabolism.^153^ Moreover, MM contains mitochondria genetic material, mitochondrial DNA (mtDNA), which encodes mitochondrial proteins for ATP production. Mutations of mtDNA often cause mitochondrial dysfunction, ultimately resulting in a devastating array of mitochondrial diseases.^154^ Obtaining a comprehensive understanding about the relationship between mitochondrial structure, function, and biochemical activity will promote the development of therapeutic modulation based on mitochondrial dynamics.

### Consequences of mitochondrial dysfunction

As a double-edged sword, mitochondria not only generate the majority of cell chemical energy, but they are also critical modulators of programmed cell death (Fig. 4b). Dozens of death signaling pathways are localized in the mitochondria which exert lethal functions in pathological conditions.^155^

Mitochondria, as essential regulators, control the activation of the intrinsic apoptosis pathway. The mitochondrial outer membrane permeabilization (MOMP) represents a critical event during intrinsic apoptosis.^156^ Several factors have been identified that can contribute to mitochondrial permeability transition pore (mPTP) opening, such as signal transducers protein P53, AKT kinase activating protein BH3, pro-apoptotic factors (Bax, Bak, Bid and Bad) or anti-apoptotic factors (Bcl-2, Bcl-XL, Mcl-1).^157,158^ MOMP directly leads to the release of apoptotic factors (cytochrome *c*, Smac/Diablo, Omi/HtrA2) from the mitochondria and into the cytoplasm. Further multimeric apoptosome is recruited when cytochrome *c* binds to APAF1, and activates pro-caspase 9.^144,155^ Subsequently, executioner caspase 3 and 7 are activated, initiating a caspase cascade for cancer cells apoptosis.^159^ Of note, MOMP and cytochrome *c* release are feature points of intrinsic apoptosis.

Mitochondria also play a crucial role in non-apoptotic cell death, particularly in mitophagy and necroptosis. Mitophagy refers to the process of the degradation or elimination of dysfunctioning, impaired or depolarized mitochondria, for maintaining homeostasis of the intracellular environment and normal cellular function.^160^ Increased evidences have indicated that mitophagy suppresses metastatic growth in the early stage of cancer and promotes advanced cancer survival.^161,162^ As an emerging target, mitophagy is available for invasive cancer treatment.^163^ Necrosis is always considered as an accidental, uncontrolled, and highly inflammatory form of cell death.^144,164^ However, some studies pointed out that its occurrence may be regulated. In some circumstances, necrosis is closely related to mitochondrial dysfunction, such as reactive oxygen species (ROS) over-generation and ATP depletion, also termed necroptosis.^165^

As a non-apoptosis, pro-inflammatory, and caspase-independent cell death modality, ferroptosis is regulated by the lethal accumulation of iron-dependent lipid peroxides.^166^ Under normal conditions, most iron is sequestered into iron-binding proteins and controlled by glutathione peroxidase 4 (GPX4) and glutathione during utilization.^167^ Iron is required in vital processes, such as respiration and DNA synthesis, and acts as a co-factor in the Fenton reaction to generate highly reactive hydroxyl radicals.^41^ Nevertheless, excessive iron loading leads to oxidative damage through the Fenton reaction, killing the cell by attacking lipid bilayers of membranes.^134^ Recently, studies have shown the relationships between mitochondria and ferroptosis. Gao et al.^41^ found that MMP hyperpolarization is related to cysteine-deprivation-induced (CDI) ferroptosis. In addition, Fang et al.^168^ observed that mitochondria-targeted antioxidants (Mito-TEMPO) enable the suppression of DOX-induced ferroptosis-induced heart damage. These findings support the effect of the mitochondria on ferroptosis. While great progress has been made in this area, studies of mechanisms and relationships between ferroptosis and mitochondria are still in their infancy; much remains to be investigated. Unraveling the relationship between mitochondria and cell death will inform the design of treatment solutions for improved cancer therapeutic effects. More importantly, further development of therapeutic agents which target mitochondrial-mediated cell death pathways will be expected to cure difficult-to-treat tumors.

### Intervention of mitochondria to control cancer cell fate

In 1956, Otto Warburg first proposed that the mitochondrial respiration defect has crucial involvement in cancer pathophysiology.^38^ Multiple hallmarks of cancer have been associated with mitochondrial dysfunctions, such as unlimited proliferation, anabolism enhancement, and apoptosis pathway impairment.^169,170^ Mitochondrial DNA (mtDNA) mutations have been reported in various cancers.^170^ The reprogrammed metabolism negatively affects mitochondrial metabolism for facilitating adaption of cancer cells to tumorigenic microenvironment.^171^ Therefore, mitochondria represent a promising target for eradicating cancer cells (Table 1). In general, mitochondrial membrane potential (MMP) loss, MPTP opening, and MOMP trigger proapoptotic protein released from IMS, which promotes apoptosmone formation and caspase cascade reaction activation, resulting in cell apoptosis.^172^ Herein, we will introduce a series of intervention mechanisms that cause mitochondrial structure and function abnormalities (Fig. 4b).

#### Metabolism disruption

As vital organelles for energy generation, mitochondria can convert glucose, fatty acids, and amino acids to adenosine triphosphate (ATP), which rely on interwoven complex biochemical processes, including oxidative phosphorylation (OXPHOS), the tricarboxylic acid (TCA) cycle, and β-oxidation.^173^ Distinct from normal cells, cancer cells rely on aerobic glycolysis as the predominant energy source, known as the Warburg effect.^174–176^ The intermediate metabolites during aerobic glycolysis, nucleotides, lipids, and amino acids, satisfy the energy demand of cancer cells for rapid growth and proliferation.^177,178^ Additionally, tumor migration, invasion, and metastasis are more prone to develop due to glycolysis, creating a tumor microenvironment with acidification and hypoxic.^179,180^ Metabolism remodeling directly drives anti-apoptosis occurrence of the most aggressive malignant tumors. Therefore, metabolism interference can help promote cancer cell apoptotic.

As demonstrated by several studies, multiple isoforms of pyruvate dehydrogenase kinases (PDKs) are universally over-expressed in cancer cells, resulting in pyruvate dehydrogenase complex (PDC) inactivation and OXPHOS compromise.^181–183^ PDKs are thus defined as the essential target for inhibiting glycolysis from rearranging metabolic pathways and, subsequently, the cell death.^184^ Kolb et al.^185^ constructed a mitochondrial-targeting system (Mito-DCA) to inhibit glycolysis by impeding PDK1 function. The orphan drug dichloroacetate (DCA) and lipophilic triphenylphosphonium cation (TPP) were selected as mitochondrial kinase inhibitors and mitochondrial-targeting factors. Mito-DCA can enhance therapeutic efficacy by reversing the glycolytic phenotype of cancer cells. Another type of glycolytic inhibitor, 3-bromopyruvate (3-BP), can block the function of hexokinase and glyceraldehyde 3-phosphate dehydrogenase (GAPDH), which are involved in the glycolytic process, and ultimately induces apoptosis of cancer cells.^186,187^ Liposome nanoparticles have been developed for site-specific, local delivery of 3-BP, minimizing side effects such as hepatotoxicity as well as being appliable to additional aerobic glycolysis-targeting drugs.^188^

Strong evidences have indicated that glycolysis might serve as the essential target to enhance therapeutic effects. As of now, there are 46 anti-cancer drugs with glycolysis targets, including 3-BP and DCA, that have entered clinical development or clinic translation (Table 2).^189^ Therefore, regulating glycolysis-related pathways would help develop glycolysis inhibitors to achieve suppression of tumors, which will usher in a new dawn in the age of cancer treatment.

#### Redox state imbalance

The majority of ROS by-products are generated in mitochondrial respiration.^190,191^ During this process, approximately 2% of oxygen is converted to ROS precursors, such as superoxide anion radical. Nevertheless, if not detoxified, intracellular ROS may cause disturbances in mitochondrial functions (when over a critical threshold), such as MPTP, MOMP, and mtDNA damage.^192–195^ Under these circumstances, an imbalance of intracellular ROS results in irreversible cell apoptosis. Moreover, ROS, as the signaling molecules, can initiate the signaling path of proliferation and promote the formation of blood vessels, which are essential for developing distant metastases of malignant cells.^196^ Therefore, many aberrant proliferative cancer cells are characterized by elevated levels of ROS relative to the antioxidant level of a system, termed oxidative stress. High levels of oxidative stress render cancer cells more vulnerable to the effects of exogenous substances, which can cause an imbalance in redox homeostasis.^197^ The redox state of mitochondria is a tempting target for the efficient treatment of cancer because mitochondria are susceptible to damage from oxygen radicals.^198,199^

Currently, several photosensitizers as well as therapeutic agents have entered clinical trials, such as β-lapachone (ARQ 501), menadione (2-methyl-1,4-naphthoquinone), and motexafin gadolinium, which participate in redox cycles in the respiratory chain to trigger excess generation of ROS for cancer treatment (Table 2). However, the excess endogenous antioxidant GSH in cancer cells scavenges ROS, making it very difficult to accumulate up to toxic levels.^170,196^ A mitochondrial oxidative stress amplifier was designed by Liang et al.^200^ Specifically, mitoCAT-g, supported by carbon dots loaded with atomically gold atoms (CAT-g) and conjugated with mitochondrial-targeted agent TPP and ROS generation agent cinnamaldehyde (CA) was investigated for their cancer treatment capabilities. Intracellular GSH was depleted due to covalent Au-S bonding generated between atomic gold and GSH. Therefore, ROS-mediated damage may occur once CA generated abundant amounts of ROS. MitoCAT-g drives the alteration of mitochondrial membrane potential (MMP) by modulating oxidative stress, leading to mitochondrial dysfunction, and ultimately resulting in cell apoptosis.

#### Perturbation of mtDNA

Mitochondrial DNA, or mtDNA, consists of circular double-stranded DNA with a length of 16.6 Kb,^201^ which is indispensable during the biochemical process of energy production and metabolism, primarily responsible for encoding polypeptides of the respiratory chain. The encoding of 22 transfer RNAs (tRNAs) and 2 ribosomal RNAs (rRNAs) associated with mitochondrial proteins is inseparable from the participation of mtRNA.^195,202,203^ Indeed, mtDNA is more susceptible to oxidative damage than nuclear DNA (nDNA) due to the lack of histone protection and inefficient DNA repair capacity; thus, mtDNA has an extremely high mutation frequency.^204,205^ Mutations and deletions of mtDNA lead to mitochondrial dysfunction and can affect the electron transfer of the respiratory chain and the efficiency of ATP production, resulting in the dysregulation of cell proliferation and differentiation and enhancing the risk of carcinogenesis.^206,207^ Therefore, repairing and/or degrading mutated mtDNA are crucial for improving patient prognosis and therapeutic outcomes.

Small interference RNA (siRNA), or exogenous therapeutic DNA, have been used to regulate mtDNA expression for tumor suppression.^208,209^ However, these free therapeutic genes could not achieve endosome/lysosome escape and mitochondrial localization. Weissig et al.^210^ designed a mitochondrial-targeted DQAsome vehicle to target delivery plasmid DNA (pDNA), where pDNA-mitochondrial leader sequence (MLS) peptide was loaded into mitochondriotropic cationic “bola-lipid”-based vesicles to form DQAplexes-DNA complexes (DQAplexes). DQAplexes could escape from endosomes and further selectively release pDNA to the site of mitochondria, achieving the goal of therapeutic transgenes to express into mitochondria.

### Smart design of mitochondria-targeted nanosystems

As mitochondria function is closely associated with cell death, mitochondria-targeted therapeutic agents represent a promising approach to eradicate cancer cells.^211–214^ However, unlike nuclei, mitochondria are highly impermeable organelles, where the transport and permeation of therapeutic agents are challenging due to the double-membrane architectures.^191,215^ The IMM has a complex structure composed of more than three times the proteins/lipids compared to cell membranes.^216^ Additionally, oxidative phosphorylation that occurs in IMM can cause large MMP, usually between −160 mV and −180mV.^217^ The complex IMM structure with high negative membrane potential and hydrophobicity render it difficult for macromolecules and bioagents to transport through the IMM for reaching the MM.^218^ There is a strong need for developing mitochondrial-targeted nanoplatforms which can satisfy the key requirements of cancer treatment.

#### Delocalized lipophilic cation

The large, negative membrane potential and high lipid content of mitochondria collectively favor selective transportation and mitochondrial accumulation of lipophilic cations. Commonly used delocalized lipophilic cations (DLCs) include triphenylphosphonium (TPP), aedualinium (DQA), berberine (BBR), rhodamine, and cyanine dyes.^154,219,220^ Among these molecules, TPP acts as the representative DLC, which is commonly used in mitochondrial-targeted nanosystem construction.^221^ According to the Nernst equation, TPP enables passage rapidly through the mitochondrial membrane and achieves almost 1000-fold accumulation in the mitochondria, driven by the MMP (at −180mV) and hydrophobic effect.^222^ Thus, TPP can play an instrumental role for mitochondrial-targeted therapeutic vehicle construction in malignant cancer treatments.^214,220,223^

DLC (as mitochondrial-targeted agents) has been widely employed in constructing various biomolecule probes and therapeutic agents.^224^ At high concentrations, it stimulates or even induces cytotoxicity against mitochondria which results in cell death. Underlying toxicity from DLCs primarily involves inhibiting F0F1-ATPase, limiting the activity of a respiratory enzyme, interference with mtDNA, and/or inducing mitochondrial membrane depolarization. These phenomena can cause mitochondria dysfunction and decreasing ATP generation.^218,223^ Additionally, the use of DLC is limited by the polarity of cargoes. DLC acts as well-investigated carriers of lipophilic or small polar molecules, yet exhibiting unsatisfactory efficiency in large polar molecule mitochondrial transportation.^144^

#### Peptide

An emerging strategy to target mitochondria is using peptide-based nano-systems, in which the sequence or the structural motif of the peptide could be rationally designed depending on the needs, compared with the DLC system. Inspired by cell-penetrating peptides (CPP), Horton et al.^225^ first designed a mitochondrial penetrating peptide (MPP) and confirmed its promotion of cell internalization and intra-mitochondrial localization. Among them, methylated lysine (K), arginine (R), phenylalanine (F), and cyclohexylalanine (Fx) were selected as the MPP units in order to respond to the unique lipophilic characters and negative potential of mitochondrial membranes. Localization analysis in HeLa cells demonstrated that MPP exhibited excellent mitochondrial localization and facilitated mitochondrial membrane fusion, further corroborated by additional studies with similar results.^226^ MPP exhibits excellent mitochondrial-targeted ability and protects mitochondrial anoxia from damage, as well as provide great potential in mitochondrial-targeted nanoplatforms design. MPP, with expected pharmacokinetic profiles, is currently undergoing active development focused on mitochondrial-related diseases.

In addition to MPP, Szeto-Schiller (SS) peptides, XJB peptides, and ATAP peptides are also used for mitochondrial-targeted nanoplatform construction. SS peptides were initially developed as antioxidants for reducing ROS generation and inhibiting mitochondrial permeability transition.^227^ Later, SS peptides were observed to cross the IMM based on the electrostatic interactions to achieve mitochondrial accumulation. SS-31 (D-Arg-Dmt-Lys-Phe-NH 2) is a SS peptide utilized for ischemic brain injuries by scavenging the toxic ROS, reaching phase II trials. XJB-5-131 peptide (Leu-D-Phe-Pro-Val-Orn) is a derivative of gramicidin S.^37^ Unlike other peptides, XJB-5-131 peptide can enter the IMM, rather than relying on MMP to achieve mitochondrial localization.^144,228^ Mitochondrial-targeting peptides are an intriguing platform for allowing structure design and biopharmaceutical function by manipulating the subsequence of a peptide.

#### Mitochondrial targeting sequence

In mitochondria, 98% of proteins are encoded from the nuclear genome and synthesized in the cytoplasm, which are then translocated to different compartments of mitochondria.^229^ Notably, highly-efficient migration of the precursor proteins to mitochondria depends on an N-terminal or C-terminal mitochondrial targeting sequence (MTS).^230^ MTS primarily includes the N-terminal sequences and tail-anchored sequence composed of a positively charged and hydrophobic stretch of 20–40 amino acid residues, so MTS possesses a hydrophobic surface containing positive charges.^144,231,232^ Evidences have been presented that MTS could be recognized by the mitochondrial import protein and further inserted into the OMM and IMM, or undergo interactions with the mitochondrial protein import complex, which ultimately achieve translocation across the mitochondrial membranes.^36^ Moreover, it is worth noting that MTS exhibits broad applicability in transporting various polar molecules. Therefore, it is important to select site-specific mitochondrial-targeted MTS for target-specific therapies, according to the heterogeneity of the disease. While MTS can exhibit excellent biocompatibility, MTS-cargo transportation is limited by the MTS transport channel size in the IMM and OMM to a certain extent.^233^ Cardiac cells, for example, allow NP transport through the OMM only when sizes are below 3 nm, while the IMM restricts NPs with sizes greater than 2 nm.^233,234^ Therefore, MTS faces stringent cargo size limitations. As such, developing versatile nano-systems with varying shapes/sizes, can provide a promising alternative solution to meet the need of specific mitochondrial compartment localization. Fortunately, increases in DLC-mediated MMP and peptide-mediated membrane fusion promote macromolecular translocation in mitochondria. It is essential to select the most appropriate correlation of mitochondrial-targeting agents, according to the cargoes unique physio-chemical properties and the reaction site-specific targets (IMM, OMM, and IMS), which can maximize the treatment efficacy.

## Lysosomes-targeted strategies-twisting cell suicide switch

### Structure of lysosome

**Fig. 5:**
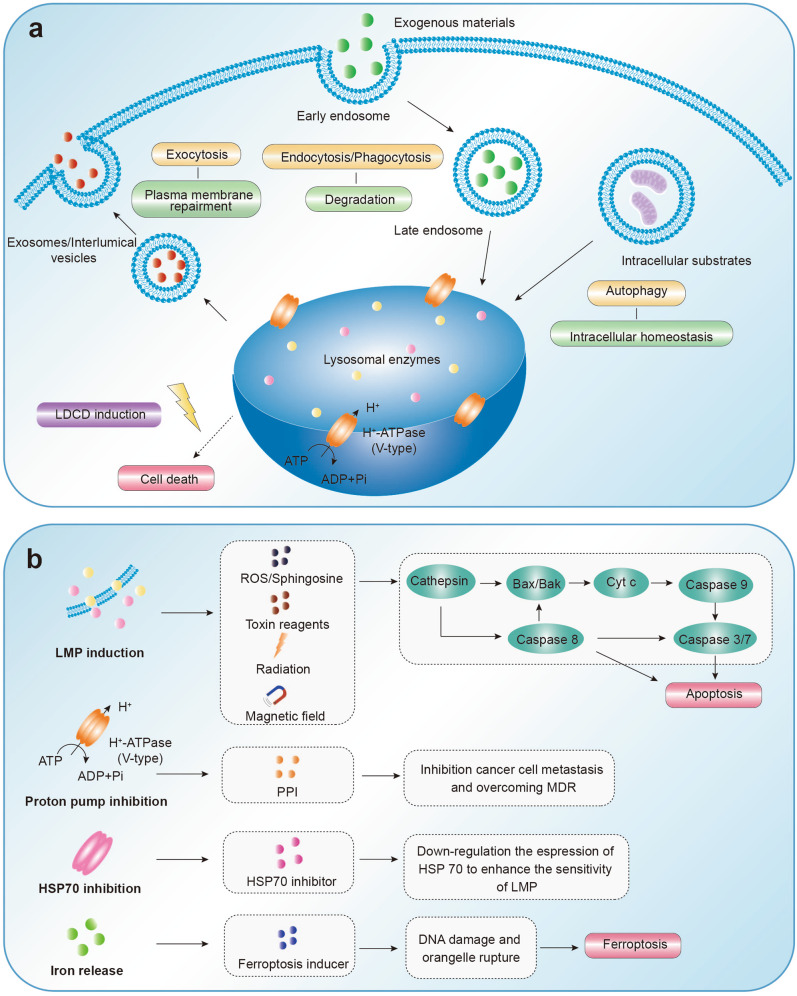
The personalized therapeutic strategy toward lysosomes. **a** Lysosomes play a vital role in exocytosis, endocytosis, autophagy, and cell death. **b** LMP induction, as a typical approach, can be triggered by ROS, toxin reagents, radiation, and magnetic fields, eventually leading to caspase-dependent cell death. Proton pump inhibition is another strategy that enables overcoming MDR. Furthermore, HSP70 inhibition and iron release increase sensitivity to lysosomal-dependent cell death (LDCD). LMP lysosomal membrane permeabilization, ROS reactive oxygen species, Cyt C cytochrome c, PPI proton pump inhibitors, HSP 70 heat shock protein 70

The lysosome, known as the “suicide bags” of the cell, were first described by Christian de Duve in 1955.^43^ This simplified understanding of the organelle has deeply evolved since, and now it is perceived as a crucial component in degrading and recycling cellular waste (Fig. 5a).^235^ Broadly speaking, lysosomes are spherical or ellipsoidal, which is no more than 1 μm in size with primarily perinuclear distribution. The shape, size, and quantity of these features vary largely depending on the cellular state and cell type.^236,237^ Lysosomes are single membrane-enclosed vesicles composed of a 7–10 nm phospholipid bilayer, containing a unique acidic lumen with a pH of 4.5–5.0.^238,239^ The acidic lumen is an integrated system maintained by proton pump V-ATPases, ion channels, and membrane transport proteins, to collectively provide an optimal environment for the degradation of hydrolytic enzymes.^240,241^ Up to now, almost 60 hydrolytic enzymes have been found in lysosomes, including sulfatases, proteases, phospholipases, and phosphatases. They can participate in autophagy and process the digestion and recycling of macromolecules, organelles, and exogenous substances to remobilize nutrients and maintain cellular homeostasis.^242,243^

Furthermore, lysosomes are inseparable from various essential processes including plasma membrane repair, mitogenic signaling, energy metabolism, immune responses.^244–246^ Lysosomal function defects impose a heavy burden, with approximately 50 monogenic diseases associated with lysosomal dysfunction, such as lysosomal storage disorders (LSDs) caused by mutations of lysosomal proteins.^247^ Thus, lysosomes are of fundamental physiological importance in cell life activities and are anticipated to be an emerging target for multiple diseases.

### Lysosome responding to cell death

Lysosomes, the vital command-and-control organelle for cellular metabolism and signaling, is associated with cell survival and death, including apoptosis, necrosis, and autophagy.^238,248,249^ It has been reported that lysosomes can stimulate cancer cell invasion, angiogenesis, and drug resistance, correlated with poor prognosis. Even though lysosomes increase the tumorigenic potential of cancer, they are more fragile, with higher instability and sensitivity to the death of cancer cells.^250^ In some particular situations, lysosome-mediated cell death programs (initiated with hydrolytic enzyme release) are termed lysosomal-dependent cell death (LDCD).^251^ The two-sided effects primarily depend on the location of lysosomal enzyme release, which is related to the process of lysosomal membrane permeabilization (LMP) and exocytosis (Fig. 5b).^252^ Intracellular released cysteine cathepsins result in cancer cell diminishment, whereas they are pro-oncogenic if extracellulary released as they then promote angiogenesis and migration of cancer cells.

Compared with a normal cell, lysosomes of cancer cells exhibit a stark difference in volume, number, and distribution, which are strongly associated with carcinogenesis.^253,254^ On average, cancer cells express lysosomes near the plasma membrane about three times as much compared to normal cells. Additionally, the increased expression of lysosomal hydrolases is a widespread phenomenon in the majority of cancer cells, related to the poor prognosis of tumors. Previous studies noted that the expression of cathepsins is upregulated in cancer cells. The extracellular mis-localization of lysosomal cathepsins stimulates tumor angiogenesis, thus promoting tumor growth, invasion, and metastasis.^255,256^ Moreover, sphingosine kinase SK23–25 is overexpressed in tumor cells, while acid sphingomyelinase is downregulated, causing the disordered sphingolipid metabolism to affect lysosomal function and membrane structure and increasing lysosomal biogenesis.^256–260^

Abnormal lysosomes increase the tumorigenicity potential, whereas lysosomes with thinner membranes and enlargement volumes can be de-stabilized in cancer cells, increasing cell death sensitivity.^242^ One critical process that is closely linked to the LDCD is LMP.^250^ LDCD is triggered by the leakage of hydrolytic enzymes into the cytoplasm, predominantly hydrolases, leading to a series of responses that are associated with cell death, such as chromatin condensation, DNA fragmentation, phosphatidylserine exposure, plasma membrane blebbing, and aberrant degradation of cellular components.^261^ The releasing extent of cathepsin into the cytoplasm determines cell death mechanisms, such as apoptosis and/or necroptosis.^246^ Executioner caspases are activated by the moderate release of cathepsin, transmitting a complex signaling cascade that eventually results in LDCD. In contrast, a massive release of lysosomal cathepsins can lead to cell necrosis due to the damage to the lysosomal membrane. Additionally, lysosomal calcium release plays an essential function in this process. Thus, lysosomal membrane integrity is critical for maintaining cellular homeostasis and regulating cellular physiological functions.^262^

Moreover, during apoptosis, lysosomes could interact with mitochondria.^242,263^ After oxidative stress, low concentrations of hydrogen peroxide drive LMP before inducing mitochondrial dysfunction. Mitochondrial dysfunction causes overproduction of ROS and impairs lipid metabolism, eventually triggering LMP. Ultimately, lysosomes play integral roles in initiating and executing cell death.

### Future targeting to lysosome for intervention

Lysosomes are crucial organelles that participate in extensively crucial cellular processes.^250^ Intervention targets of the biochemical pathways mediated by lysosomes have been demonstrated as innovative therapeutic strategies that can induce programmed cell death (Table 1 and Fig. 5b).

#### LMP induction

LMP has been demonstrated to be an effective strategy to trigger LDCD,^263^ where massive lysosomal leakage can cause cytoplasmic acidification and uncontrolled degradation of cellular components leading to potential cell death. Indeed, lysosomes in cancer cells are more vulnerable to LMP due to oncogenes downregulating lysosomal membrane protection proteins, which are highly glycosylated glycoproteins.^264^ Additionally, hydrolysis of sphingomyelin, where lysosomal membranes are rich in, sensitizes cancer cells to LMP.^258,265^ Cancer cells with enlargeable lysosome size and number are thus more vulnerable to LMP-mediated apoptosis.

Among various external and internal stimuli, intracellular second messengers (ROS and sphingosine), lysosomal toxin reagents, and radiation primarily contribute to lysosome instability and disrupt the lysosomal integrity, which can cause pore-formation and LMP initiation.^245,266^ Additionally, LMP induction by magneto-mechanical effect of particles (TMMEP) is an emerging research area. The magnetic vibrations of these nanoparticles, induced by a mechanical force, leads to cancer cell destruction.^267^ Cheng et al.^268^ synthesized highly-magnetized, zinc-doped iron oxide nanoparticles to mechanically destroy cancer cells at low frequency by rotating magnetic fields (15 Hz and 40 mT). Lysosomal membrane integrity is disrupted by the magnetically anisotropic aggregates, leading to LMP-induced cell death. Moreover, iron oxide nanoparticles are also widely used to initiate lysosomal permeabilization at pulsed magnetic fields.^269^ Harnessing LMP emerges as a primary strategy for constructing the lysosomal-targeted therapeutic agents. Given the diverse strategies available for inducing LMP, a method that efficiently destroys lysosomes is promising for eliminating damaged cells. As such, a key objective of LDCD will be a better understanding of the LMP mechanism and LMP-inducing agent action.

#### Proton pump inhibition

The vacuolar H^+^-ATPase (V-ATPase), an evolutionarily multi-subunit complex, acts as proton pumps responsible for regulating the acidic environment of the intracellular, acidic lumen of lysosomes, and extracellular space.^270^ The acidic environment of the lysosome is primarily maintained by V-ATPase pumping protons into the lysosomal lumen. However, abnormalities in the V-ATPase proton pump promotes intracellular alkalinization and extracellular acidification processes, which are commonly observed in invasive tumors.^271^ More importantly, the V-ATPase proton pump also significantly impacts the multidrug resistance (MDR).^272,273^ In particular, weakly basic anticancer drugs (such as anthracyclines) are prone to protonation in acidic environments. The drug entering the cytosol is hindered by accumulation in lysosomes following protonation, thus leading to drug resistance.^274,275^ MDR cancer cells usually exhibit V-ATPase activity enhancement, which treatment can be further complicated.^276^ Therefore, regulating V-ATPase activity may enhance the chemosensitivity of MDR cancer cells toward chemotherapeutic drugs.

In recent years, much attention has been focused on targeting tumor acidity and improving the microenvironment to inhibit cancer cell metastasis and reverse MDR.^277–279^ Unlike conventional cytotoxic anticancer drugs, proton pump inhibitors (PPI) target tumor microenvironments to achieve efficient tumor killing.^277^ Of which, pantoprazole, omeprazole, and lansoprazole have been confirmed to exhibit efficient anti-cancer activity by suppressing cell viability and metastasis, facilitating cell apoptosis (Table 2). Moreover, PPI participates in a complex biological process that modulates cancer progression through protein-protein interactions and various signaling pathways.^277^ However, long-term PPI usage can lead to serious side-effects which may affect nutrient absorption and lead to complications, enhancing the incidence of cancer through heterogeneous tumors.^280^ Consequently, further investigations about the action mechanism of PPI is necessary to determine a more precise action mode with lesion targets; proper caution is imperative, regarding adverse effects when treating cancer cells with PPI.

#### HSP70 inhibition

Several small molecules, such as heat shock protein 70 (HSP70), have been identified as lysosome membrane stabilizers, which can prevent LMP.^254^ The overexpression of HSP70 in cancer cells improves the resistance of membrane instability enhancing cell survival.^281^ HSP70 binding to LMP-inducing factors (such as p53) limits the membrane rupture or dysfunction.^282,283^ Moreover, HSP70 interacts with bis-monoacylglycerol-phosphate (BMP), forming Hsp70-BMP to improve sphingolipid hydrolysis and eventually promoting the stability of lysosomal membranes.^281,284^ The suppression of HSP70 function is therefore an emerging target for cancer therapy.^285,286^ Applying HSP70 inhibitors (2-phenylethynesulfonamide, PES) or inhibiting the related regulators of HSP70 expression (such as heat shock factor 1, HSF1) to down-regulate the expression of HSP70 is a well-recognized entity for enhancing the sensitivity of LMP in cancer cells.^287,288^

#### Iron release

Iron is the most abundant transition metal in the human body, playing a vital role in the human health.^289,290^ Specifically, iron participates in many biological processes, such as electron transport, enzymatic reactions, oxygen transport, and DNA synthesis.^291^ Previous studies have noted iron concentration discrepancies between normal and cancer cells.^292^ Lysosomes accumulate a significant portion of iron with redox activity due to the degradation of iron-containing metalloproteins.^293^ Once excessive iron in the labile iron pool is released to the cytoplasm, it can act as a pro-oxidant factor contributing to excess ROS generation based on the iron-catalyzed Fenton reaction. Subsequently, a range of biological responses can occur, such as DNA damage and organelle rupture, also termed ferroptosis cell death.^294–296^ Therefore, developing efficient methods for inducing ferroptosis cell death is important for lysosomal targeting cancer treatment.

### Lysosome-targeted nanosystems design

Lysosome-targeted treatment strategies significantly contribute to the development enhanced cancer therapy, as the accumulation of therapeutic nanoplatforms within the lysosome are more accessible than in other organelles.^297,298^ Studies have confirmed that exogenous cargo modified by a specific ligand or by optimizing with specific physicochemical properties could be internalized by cells upon receptor-mediated endocytosis, and eventually accumulated in lysosomes.

The intercellular internalization pathways of cell surface components and extracellular macromolecules primarily involve clathrin-dependent endocytosis (such as receptor-mediated endocytosis) and clathrin-independent endocytosis (phagocytosis, micropinocytosis, and caveolin-mediated endocytosis).^299^ One of the most well-characterized forms of endocytosis is the receptor-mediated endocytosis, also referred as RME, which is responsible for cellular internalization between specific ligands and cell surface receptors.^300–302^ During endocytosis, the plasma membrane invaginates to form luminal vesicles that are then fused with endosomes to enter the endolysosomal membrane system.^303^ The extracellular materials eventually arrive in specific lysosomal locations under the endocytosis pathway. Therefore, after modifications with specific receptors, the therapeutic agents can enter lysosomes from the extracellular environment by interacting with a high-affinity ligand on the surface of cancer cells.^304,305^ As a result, active-targeting receptor-mediated endocytosis may be a promising strategy to achieve accumulation in lysosomes.

The physicochemical properties of nanoplatforms (such as size, charge, and flexibility) significantly impact lysosomal retention. Human HT29 colon cancer and SKB3 breast cancer cells which express chimeric receptors were utilized as a model to investigate the endocytosis efficiency of size- and rigidity-dependent nanoparticles.^306^ The internalization rate of larger and more rigid nanoparticles was found to be much slower than that of smaller nanoparticles. In general, cationic nanoparticles can penetrate the cell membrane barrier more efficiently than anionic nanoparticles due to the positively charged surfaces favor electrostatically interactions with the negative charges of cell membranes.^307–309^ Furthermore, cationic nanoparticles induced membrane depolarization, resulting in membrane permeabilization that ultimately contributes to cell death.^310,311^ A type of mixed-charge nanoparticle was constructed through reasonable regulation of positively and negatively charged ligand ratios by Borkowska et al.,^312^ termed [+/−]NPs, which could selectively target lysosomes with improved cell internalization efficiency accompanied with negligible cytotoxicity to normal cells. The [+/-]NPs induced lysosomal swelling and disrupted lysosomal integrity, ultimately triggering the death of cancer cells.

Additionally, lysosome-targeted fragment modification is another strategy that has been applied to achieve nanoplatform accumulation within lysosomes. Alkylated piperidine fragments are trapped within lysosomes as they protonate in an acidic environment, which can then be used as targeting factors.^313^ Daum et al.^313^ designed a novel prodrug based on lysosome-targeting ROS amplifiers. Specifically, *N*-alkylaminoferrocene was modified with an alkylated piperidine fragment to achieve lysosome targeting. The prodrug was activated by high ROS concentration in lysosomes, eventually disrupting the cell cycle by attacking lysosomes and disrupting ROS balance. *N*,*N*-dimethylpropane-1,3-diamine could also be used for lysosome-targeting with fluorescent chemosensor (Lyso-HS) modification. The tertiary amine of Lyso-HS can be protonated under the lysosomal microenvironment, and thus Lyso-HS remains in the lysosome and allows for H_2_S detection.^314^

## ER-targeted strategies-a perturbation site of protein homeostasis

### Structure of ER

The ER is one of the largest and most complicated intracellular organelles, spanning from the outer NE up to the boundary of the cell membrane.^315,316^ Depending on the dynamic membranous network of tubules, lamellae, and vesicles, the ER communicates with various cellular organelles, including the mitochondria, Golgi apparatus, and cell membrane, and facilitates protein and lipid transport between various compartments.^317,318^ This important organelle is the central hub for protein folding and processing, lipid and sterol biosynthesis, and intracellular calcium storage and buffering.

The ER lumen contains a protein quality monitorization system that modulates the correct folding and complex formation of expressed proteins,^319,320^ where only correctly folded polypeptides are delivered to their destination following release from ER. Almost 30% of nascent proteins are folded in the ER lumen with the assist of a series of molecular chaperones.^321^ Unfolded or misfolded proteins can trigger unfolded protein response (UPR) signaling pathways to transport them out of ER and to subsequent degradation by the proteasome.^322^ If unfolded or misfolded proteins are not promptly removed, perturbations of ER homeostasis can lead to severe ER stress.^323^ A series of diseases, such as diabetes mellitus, Alzheimer’s disease, many cardiovascular conditions, and inflammation-related diseases, have been found to be linked to overactive ER stress.^324–326^ More recently, mounting evidence suggests that UPR plays a critical role in the survival and maintenance of cancer cells.^327^ More importantly, as a Ca^2+^ storage compartment, the ER regulates the equilibration of intracellular Ca^2+^ homeostasis.^328^ In general, resting cytosolic Ca^2+^ concentration is between 50–100 nM, which is significantly lower than the 100–800 μM in the ER.^329^ Indeed, high Ca^2+^ concentration in the ER is a requisite for the functioning of ER chaperones,^330^ which is also essential for maintaining an oxidizing environment in ER lumen to promote disulfide bone formation during protein processing.

### Unfolded protein response: friend or foe?

**Fig. 6:**
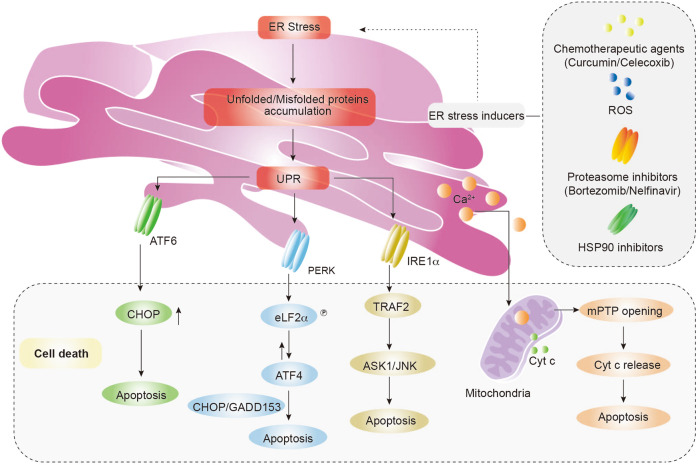
Unfolded protein response (UPR) is a valuable target in cell death. Protein misfolding or unfolded can occur as a disturbance in ER homeostasis, leading to ER stress. Chemotherapeutic agents, ROS, proteasome inhibitors, and HSP 90 inhibitors as ER stress inducers perturb ER homeostasis differently. If ER stress is not resolved in a timely fashion, unfolded or misfolded proteins accumulate in ER, and UPR triggers cell death via ATF6, PERK and IRE1α mediated signaling pathways. Importantly, fluctuations in ER and mitochondrial Ca^2+^ homeostasis can initiate mitochondrial-mediated cell death. UPR unfolded protein response, ROS reactive oxygen species, HSP 90 heat shock protein 90, mPTP mitochondrial permeability transition pore, CHOP C/EBP homologous protein, ATF6 p50ATF60, ATF4 transcription factor 4, TRAF2 TNF receptor-associated factor 2, ASK1 apoptosis signal-regulating kinase 1, JNK c-jun N-terminal kinase

Many studies indicated that the ER plays a pivotal role in initiating apoptosis. As discussed above, ER stress occurs when protein misfolds during biosynthesis. In response to ER stress, UPR is activated to address the unfolded or misfolded protein threat and re-establish normal ER function.^322,331^ In the ER membrane, three transmembrane proteins (PERK, IRE1α, and ATF6) have been recognized to ER stress and promote pro-survival pathways. However, if prolonged ER stress or UPR recovery fails, the apoptotic signaling pathway will be activated to remove damaged cells (Fig. 6).^332^

Proapoptotic protein C/EBP homologous protein (CHOP/ GADD153) regulates ER stress-induced apoptosis and promotes cell death.^333,334^ When ER stress persists, PERK phosphorylates eIF2α and subsequently activates and upregulates the expression of transcription factor 4 (ATF4), which directly triggers CHOP/GADD153 mediated ER-stress-induced apoptosis.^335^ Moreover, after activating cleavage, the ATF6 (p50ATF60) cleavage product upregulates the expression of pro-apoptosis protein, such as CHOP, and consequently induces apoptosis.^336^ Additionally, IRE1α regulates another ER stress-induced cell death pathway, where it recruits the adapter molecule TNF receptor-associated factor 2 (TRAF2) and subsequently activates apoptosis signal-regulating kinase 1 (ASK1) and c-jun N-terminal kinase (JNK), eventually leading to cell death.^337^

Likewise, Ca^2+^ in the ER plays an integral role in the ER stress-mediated cell apoptosis. While Ca^2+^ flux and leakage from the ER occur, significant amounts of Ca^2+^ can enter and accumulate in the MM along the ER-mitochondria contact sites, collapsing the mitochondrial function.^338^ Mitochondrial Ca^2+^ overloading is intimately associated with cell death, where a high concentration of Ca^2+^ can trigger mPTP opening and release mitochondrial pro-apoptosis factors to initiate apoptosis.^339^ These examples indicate the ER is crucial in deciding cell survival and death.

### Go in for the kill: how to trigger unfolded protein response?

In comparison to normal cells, cancer cells reprogram their intrinsic metabolism patterns to adapt unfavorable environments for survival and then relentlessly proliferate.^340^ A wide variety of studies have indicated that ER stress and UPR activity are directly correlated with tumor invasion, metastasis, and chemo-resistance to different types of cancer.^341–343^ ER stress and UPR overactivation are common phenotypes in most cancer cells,^344^ where UPR overactivation enables the management of protein translation to protect cells from ER stress damage, and thus, increase cancer viability under unfavorable environments.^345^ Many studies also suggest that changes in UPR component expression, such as GRP78/BIP, UPR trans-activators XBP1, and ATF6, have been detected in numerous types of human cancer.^346^ Therefore, increasing ER stress could be a potential strategy for therapeutic intervention.

Excessive ER stress causes pro-apoptosis signaling pathway activation, eventually causing cell death. Different ER stress inducers, such as chemotherapeutic (curcumin and celecoxib) and ROS, can target the ER and subsequently induce ER-stress apoptosis (Fig. 6).^347,348^ In addition, Ca^2+^ imbalance and intracellular hypoxia environments accelerate ER stress and ER dysfunction, followed by cell apoptosis.^349^

The clinical significance of UPR as a vital target has been increasingly recognized in cancer therapy.^334^ As proteasome inhibitors, Bortezomib, Nelfinavir, and Atazanavir, have been employed against prostate, lung, breast, and colon cancer in clinical trials (Table 2).^350,351^ They are involved in UPR activities, leading to misfolding protein accumulation in the ER and generating enhanced ER stress. Alternatively, HSP90, as molecular chaperones, can participate in the folding process of substrate proteins during UPR.^352^ They are frequently mutated or overexpressed in tumors to protect from ER stress damage.^353^ HSP90 inhibitors disrupt HSP90 client protein folding, such as oncogenic proteins, and eventually lead to cell death.^354^ It is worth mentioning that UPR is overactivated in cancer cells, which is not always the case in normal cells. The difference of cancer cells compared with normal cells on UPR can be exploited to reduce toxic side effects during cancer treatment.

### Strategies to achieve ER accumulation

Given that it serves several essential roles in apoptosis, targeted delivery of therapeutic agents into the ER is of significant importance for cancer therapy. However, therapeutic agents which can selectively navigate into ER is a daunting task due to the ER’s complex structure, containing a vast 3D interconnected network of different thicknesses.^355^ The ER-targeting strategy is of great clinical importance as it provides a key target for anticancer drug development and cancer treatment advancement.

#### Small molecules

ER-targeting small molecules specifically bind to the surface of the ER, accumulate, and disrupt ER function.^356^ Sulfonamide ligands have been extensively developed for small molecule drugs and drug delivery vehicle modification due to its low toxicity, high efficiency, and high selectivity.^357^ They specifically recognize and bind to sulfonylurea receptors with high affinity, which are potassium-selective ion channel proteins highly expressed on ER membranes.

Glibenclamide, a sulfonamide urea derivative, can assist commercial fluorescent probes, such as ER-Tracker Red and ER-Tracker Green, into the ER to achieve membrane visualization. Additionally, the dansyl and toluenesulfonyl groups in *N*-(2-aminoethyl)-5-(dimethylamino) naphthalene-1-sulfonamide can serve as typical sulfonyl ligands that endow therapeutic agents with ER-targeting ability.^358,359^ Basu et al.^360^ engineered 17AAG-ER-NPs with an ER targeting group (toluenesulfonyl) and HSP90 inhibitor (17AAG, ER stress inductor) to trigger ER stress-mediated cell death. This nanoplatform prompted remarkable anticancer efficacy at sub-micromolar concentration, providing a promising alternate for cancer treatment. Chen et al.^361^ synthesized polymeric, reduction-sensitive NPs, which were loaded with an ER-targeting photosensitizer containing toluene sulfonamide, to induce ER stress by local ROS generation and subsequent immunogenic cell death (ICD) activation.

#### ER-targeting peptides

ER is the primary site of protein biosynthesis, and their localization signal peptides with homing properties can assist ER molecular chaperones in delivering their duty.^362^ The KDEL peptide was first used to enhance protein accumulation in the ER in 1987, and then extensively used as an ER-retention sequence for ER recognition and localization.^363^ KDEL with the C-terminal sequence Lys-Asp-Glu-Leu motif could recognize and bind specifically to KDEL receptors (KDELR) to promote ER accumulation via a coat protein I (COPI)-mediated retrograde pathway.^364,365^ Wang et al.^366^ showed the evidence that KDEL facilitates ER transportation through monitoring trafficking pathways of KDEL-Au NPs. Interestingly, the KDEL peptide-mediated ER translocation pathway evades lysosomes to prevent degradation and protect cargos. These featured characteristics of the KDEL peptide make it e an attractive tool for ER retention of therapeutic agents in treating cell malignancies.

## Personalized therapeutic strategies toward additional organelles

### Plasma membrane

The plasma membrane primarily consists of a phospholipid bilayer structure in which various proteins and lipid species are inhomogeneously incorporated.^367^ Developing the role of the plasma membrane in cancer treatment has received significant research interests. The uncontrolled growth of cancer cells relies on plasma membrane reprogramming to satisfy rigorous requirements of biosynthesis and bioenergetics due to their rapid division.^368^ Aberrant upregulation of several lipogenic enzyme expressions, such as fatty acid synthase (FASN) and acetyl-CoA carboxylase (ACC), has been observed in many cancers, directly resulting in fatty acid synthesis and cholesterol metabolism alteration in cancer cells.^369,370^ Furthermore, an increase in sphingolipids and cholesterol content promotes abnormalities of plasma membrane permeability, contributing to reduced drug influx, P-gp efflux, and increased intracellular vesicle-mediated drug entrapment, which can trigger multidrug resistance (MDR) in drug-resistant cancer cells.^371,372^ Therefore, therapeutic strategies for plasma membranes may offer a practically feasible approach to improving treatment efficacy and enhancing the sensitivity of current anti-cancer therapies.

To enable plasma membrane-targeted personalized strategies, photodynamic therapy (PDT) has been extensively studied. Severe ROS damage to the plasma membrane may directly inhibit cell proliferation and migration, inducing apoptosis by destroying cellular integrity and activating the immune system.^373^ However, plasma membrane retention suffers from cellular uptake and endocytosis.^374^ Recently, efforts have been focused on optimizing therapeutic agent structures to prolong retention times in the plasma membrane. A pH (low) insertion peptide (pHLIP) allows for insertion into the plasma membrane spontaneously after self-transformation. A pH-driven, membrane-anchoring photosensitizer (pHMAPS), with pHLIP and protoporphyrin IX, has been designed to achieve membrane localized PDT.^375^ It was observed that pHMAPS can cause significant damage to cancer cells and significantly inhibited tumor growth, due to cytotoxic ROS generation and accumulation near the plasma membrane. Moreover, cracked cancer cell membrane (CCCM),^376^ lipophilic palmitic acid (PA),^377^ membrane fusogenic liposomes (MFLs),^378^ and cholesterol^379^ have also been applied to promote cell membrane anchoring via insertion or fusion approaches.

Notably, plasma membrane-anchoring therapies can protect therapeutic agents with significant efficacy, by safe-guarding from lysosomal degradation and retention. These strategies demonstrate another influential, promising target for precision cancer medicine. Despite their remarkable potential, these studies are still in the initial stages and more investigations are needed to improve/confirm their viability through further system optimizations, such as prolonging membrane retention time.

### Peroxisome

The origin and nature of peroxisomes have been actively debated since 1950s.^380^ It is now accepted that peroxisomes are semi-autonomous organelles which are involved in lipid metabolism regulation, fatty acids auxiliary processing, plasmalogen synthesis, and ROS modulation.^381^

Recent studies indicate that peroxisome plays a significant role in regulating cancer initiation and progression.^382^ Peroxisomes have been observed to support cancer cell energy supply by providing a lipid substrate.^383^ Alteration of enzymatic activities and protein levels related to lipid processing in peroxisomes has been identified in numerous cancer types, such as prostate, breast, liver, and ovarian cancer.^384,385^ Elevated peroxisomal fatty acids and ether phospholipids in peroxisomes help cancer cells survive various stresses, and contribute to tumor progression in an oxygen-depleted tumor environment.^386–388^

More importantly, evidence suggests that peroxisomes can cooperate with mitochondria by connecting vesicular pathways or fission machinery.^389,390^ A variety of enzymes that regulate ROS production and clearance reside both in peroxisomes and mitochondria, suggesting a possible link of metabolic cross-talk between peroxisome and ROS homeostasis.^391,392^ The loss or overproduction of Mpv17, which encodes peroxisomal proteins, directly leads to intracellular ROS production reduction or enhancement.^393^ In addition, peroxisomes play an important role in resisting ROS-mediated apoptosis and influencing cancer cell growth. Increases in peroxisome amounts were observed in vorinostat-resistance lymphoma cells against ROS damage, and further peroxisomal proteins PEX3, PEX11B, and PMP70 were also upregulated.^394^ These results indicated the role of peroxisomal participation in ROS metabolism, suggesting peroxisome metabolism may be a potential therapeutic target against cancer progression and circumvent drug resistance.

Peroxisome metabolism could be a desired target for future cancer therapeutic agents. Considering the interplay between the mitochondria and peroxisome, peroxisome disruption may rewrite metabolism pathways and have profound therapeutic effects. However, a few peroxisome activity modulators have been developed without preclinical and clinical trials. Peroxisome metabolism intervention therapies with therapeutic nanosystems are still underexplored. As such, further investigations of the peroxisome-mediated molecular pathway and the development of specific therapeutic agents are required, which may drive additional impactful cancer therapy treatments.

### Golgi apparatus

The Golgi apparatus exists as a series of flattened membrane-bound sacks (cisternae), organized in a perinuclear lace-like reticulum in a *cis*-to-*trans* fashion.^395,396^ Cellular homeostasis is highly reliant on the proper functioning of the Golgi apparatus in protein sorting and trafficking.^397^ It has been shown that Golgi glycosylation abnormalities are closely related to the occurrence and metastasis of cancers.^398^ It is thus conceivable that the fragile Golgi apparatus provides an opportunity for specific cancer-directed therapeutic approaches.

Various Golgi-disturbing agents have been developed, such as brefeldin A (BFA), monensin, nocodazole, and retinoic acid (RA), which can directly attack protein trafficking pathways mediated by the Golgi apparatus or induce ion imbalances, and subsequently, disturb the Golgi apparatus and induce apoptosis.^399–401^ Ma et al.^402^ constructed chondroitin-modified lipid nanoparticles (CSNs) to deliver RA and DOX. DOX+RA-CSNs efficiently accumulated in the Golgi apparatus to damage their structures and inhibit extracellular matrix (ECM) protein production, resulting in liver cancer cell apoptosis. Aside from chondroitin sulfate (CS), a series of novel targeting ligands can anchor in the Golgi apparatus, such as cysteine derivatives (protein kinase D and galactosyltransferase), phenylsulfonamide derivatives, and aminoquinolies.^403–406^ To date, few Golgi therapeutic agents are applicable for cancer curing. Future research directions may include revealing molecular interference targets of the Golgi, exploring the mechanisms of Golgi-disturbing agents, and new targeting tags to drive the course of cancer therapy.

## Conclusion and outlook

With the rapid and intensive research and technology development across biology, medicine, and materials science, precision subcellular-targeted nanoplatforms have become an important research topic for cancer treatment across the globe. This review presents three major organelles, including the nucleus, mitochondria, lysosomes and ER, while summarizing the unique characteristics and various functions of each organelle to unveil the hallmarks and potential for these therapeutic targets. Furthermore, underlying guidelines of organelle-targeted nanoplatform constructions are discussed according to specific characteristics of each organelle. Advancing the understandings of the interplay between organelle characteristics and functions with nanoplatform construction guidelines will be essential for enabling improved organelle-targeted therapeutic agents for future oncology development.

The organelle-targeted strategy holds tremendous potential in next-generation cancer therapies, which has gradually become the primary approach for personalized cancer treatment. While the controlled delivery at the organelle level has been achieved, their adaption to current medical practice have yet to be fully exploited. Here, we provide a brief perspective on couple ongoing challenges with several potential solutions: (1) incomplete understandings of molecular pathogenesis of tumor heterogeneity lead to noticeable variation in treatment effects in different individuals with identical treatments. More in-depth studies and enhanced comprehension of aberrant cellular signaling pathways and molecular regulators on cancer are urgently needed, with particular focuses on chemotherapy and gene therapy. Investigations of the underlying intrinsic molecular regulatory mechanisms and development of novel molecular targets are essential for enabling organelle-targeted treatment for majority cancers; (2) efficacy and safety following organelle-targeted cancer treatment requires additional study. Blood clearance and retention times for targets of nanomaterials along with physiological monitoring of individuals are still lacking. Therefore, long-term tracking of safety and treatment efficacy, as well as establishing a primate experimental model are indispensable procedures, which are necessary to validate their benefits for translations to clinical applications.

Overall, this article presents a broad and comprehensive review on the topic of potential organelle-target characteristics and the underlying system design guidelines for therapeutic agent construction. We highlight the importance of organelle-targeted therapeutic strategies for precision medicine in cancer therapeutics, which will be very important for the development of emerging organelle-targeted nanomaterials and their associated future implementation. Looking forward, we believe this formidable technology holds great potential to revolutionize cancer therapy at the interface of biology, nanomaterials, and medicine.
